# New Dual Inhibitors of Tyrosyl-DNA Phosphodiesterase 1 and 2 Based on Deoxycholic Acid: Design, Synthesis, Cytotoxicity, and Molecular Modeling

**DOI:** 10.3390/molecules29030581

**Published:** 2024-01-24

**Authors:** Oksana V. Salomatina, Tatyana E. Kornienko, Alexandra L. Zakharenko, Nina I. Komarova, Chigozie Achara, Jóhannes Reynisson, Nariman F. Salakhutdinov, Olga I. Lavrik, Konstantin P. Volcho

**Affiliations:** 1 N.N. Vorozhtsov Novosibirsk Institute of Organic Chemistry, SB RAS, 9, Lavrent’ev Ave., Novosibirsk 630090, Russia; ana@nioch.nsc.ru (O.V.S.); komar@nioch.nsc.ru (N.I.K.); anvar@nioch.nsc.ru (N.F.S.); 2 Institute of Chemical Biology and Fundamental Medicine, SB RAS, 8, Lavrent’ev Ave., Novosibirsk 630090, Russia; t.kornienko1995@gmail.com (T.E.K.); sashaz@niboch.nsc.ru (A.L.Z.); lavrik@niboch.nsc.ru (O.I.L.); 3 School of Pharmacy and Bioengineering, Keele University, Hornbeam Building, Newcastle-under-Lyme, Staffordshire ST5 5BG, UK; a.achara@keele.ac.uk (C.A.); j.reynisson@keele.ac.uk (J.R.)

**Keywords:** deoxycholic acid, oxirane, thiols, TDP1 inhibitor, TDP2 inhibitor, cancer, tumor, molecular modeling

## Abstract

Deoxycholic acid derivatives containing various heterocyclic functional groups at C-3 on the steroid scaffold were designed and synthesized as promising dual tyrosyl-DNA phosphodiesterase 1 and 2 (TDP1 and TDP2) inhibitors, which are potential targets to potentiate topoisomerase poison antitumor therapy. The methyl esters of DCA derivatives with benzothiazole or benzimidazole moieties at C-3 demonstrated promising inhibitory activity in vitro against TDP1 with IC_50_ values in the submicromolar range. Furthermore, methyl esters **4d**–**e**, as well as their acid counterparts **3d**–**e**, inhibited the phosphodiesterase activity of both TDP1 and TDP2. The combinations of compounds **3d**–**e** and **4d**–**e** with low-toxic concentrations of antitumor drugs topotecan and etoposide showed significantly greater cytotoxicity than the compounds alone. The docking of the derivatives into the binding sites of TDP1 and TDP2 predicted plausible binding modes of the DCA derivatives.

## 1. Introduction

DNA damage in malignant cells is the main strategy in traditional chemo- and radiation therapy of oncological diseases. However, the resulting DNA damage can be repaired by the cancer cells, leading to resistance. Therefore, DNA repair enzymes are considered promising targets for improved anticancer therapy [1,2,3,4,5,6].

Topoisomerase, an enzyme that catalyzes changes in the topology of DNA, plays an important role in cellular reproduction and DNA organization [7,8,9,10,11]. DNA topoisomerases prevent and correct topological issues such as relaxing supercoils and untangling catenanes. This is achieved by cleaving the sugar–phosphate backbone of either one (type I topoisomerases (TOP1)) or both (type II topoisomerases (TOP2)) DNA strands. This transient break allows the DNA strand to be untangled or unwound, and at the end of these processes, the backbone chain is resealed.

Some TOP inhibitors, such as topotecan and irinotecan (TOP1 poisons) and etoposide and doxorubicin (TOP2 poisons), stabilize covalent topoisomerase/DNA complexes, preventing re-ligation. These TOP-DNA-inhibitor complexes cause cytotoxicity, as the single- and double-stranded DNA breaks lead to apoptosis and cell death [12,13,14]. These covalent complexes can be repaired by tyrosyl-DNA phosphodiesterases 1 and 2 (TDP1 and TDP2) [15], making them potential targets for increasing the efficacy of TOP inhibitors. TDP1 catalyzes the hydrolysis of adducts that are covalently bound to DNA via the 3’-phosphate moiety including the products of proteolysis of TOP1–inhibitor complexes (TOP1 peptides), while TDP2 hydrolyzes 5′-phosphothyrosine and TOP2 adducts DNA [15]. It is important to note that the TDP1 and TDP2 enzymes have overlapping functions, albeit with less efficiency [16,17]. Also, it should be noted that the number of classes of organic compounds inhibiting TDP2 is very limited [18,19].

**Figure 1:**
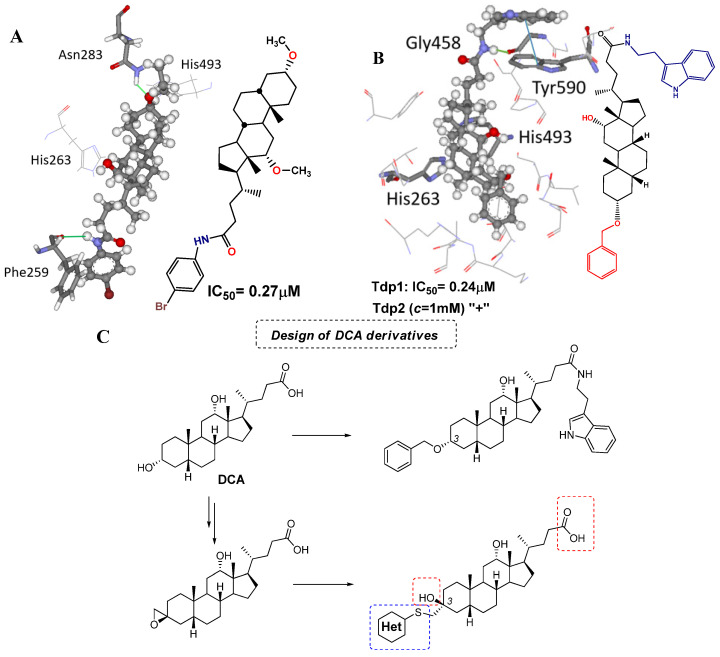
Structures of reported TDP1 and TDP2 inhibitors based on deoxycholic acid: the docked pose of 3α,12α-*bis*-methoxy deoxycholic *para*-bromoanilide (**A**) and 3α-benzyloxy deoxycholic tryptamide (**B**) in the catalytic site of TDP1. (**C**) Design of DCA derivatives as dual inhibitors of these enzymes.

We previously found that bile acid derivatives obtained via targeted modification of both the carboxyl group and the steroid backbone are effective TDP1 inhibitors [20,21,22]. The IC_50_ values of most developed deoxycholic acid (DCA) derivatives, such as 3α,12α-*bis*-methoxy deoxycholic *para*-bromoanilide (Figure 1A) as well as 3α-benzyloxy deoxycholic tryptamide (Figure 1B) [21], have been shown to be in the submicromolar range. Additionally, it was found that the 3α-benzyloxy deoxycholic tryptamide (Figure 1B) inhibited the TDP2 enzyme with residual activity less than 50% at ~1 mM concentration [22]. Moreover, an additional design challenge is the low water solubility of these inhibitors.

Herein, we continued to develop dual inhibitors of TDP1 and TDP2 with special attention toward increasing the inhibitory activity against TDP2 and improving the water solubility of the ligands.

Via analyzing the results of molecular modeling of previously obtained TDP1 inhibitors based on DCA [21,22], it can be seen that the bromobenzyl ring in 3α,12α-bis-methoxydeoxycholic acid *para*-bromoanilide (Figure 1A) occupies the same space as the benzyloxy ring fragment in the tryptamide of 3α-benzyloxydeoxycholic acid (Figure 1B), i.e., the modification of the steroid core at position C-3 with an aromatic moiety changes the mode of ligand binding. Unfortunately, for the TDP2 enzyme, we were unable to identify any significant differences in the binding of deoxycholic acid derivatives—the same binding mode is predicted for all DCA ligands, whether active or inactive [22]. Therefore, we decided to focus our attention on the modification of the third position of the DCA steroid backbone with various aromatic fragments. A significant disadvantage of benzyl ethers is their relatively high lability, which allows them to be used as protective groups, so we decided to modify the third position using an epoxy group as a key intermediate (Figure 1C). It is known that the carbonyl group at C-3 in steroid derivatives selectively reacts with the dimethylsulfoxonium methylide to form only β-oxaspiro derivatives [23]. The subsequent opening of the epoxy ring under the S_N_2 reaction conditions leads to the functionalization of the steroid backbone from the α-side, which will allow us to gain fundamental knowledge about the prospects of such an approach for the synthesis of metabolically more stable “analogs” of benzyl ethers. The proposed route allows not only the introduction of various functional groups at the C-3 position with high stereoselectivity but also the preservation of the hydroxyl group at the C-3 position. A similar strategy is widely represented in the chemistry of natural compounds [24,25,26,27,28]. As for the carboxyl group in the side chain, we decided not to modify it since the presence in the design molecules of three polar groups (two hydroxyl and carboxyl) will allow us to reduce excessive lipophilicity of the obtained inhibitors based on DCA.

In this work, we synthesized new deoxycholic acid derivatives and studied their effect on the phosphodiesterase activity of the TDP1 and TDP2 enzymes. The cytotoxic effect of lead compounds against cervical carcinoma cells of Hela has been investigated, both alone and in combination with topoisomerase inhibitors topotecan and etoposide. It has been shown that compounds modified with benzothiazole and benzimidazole moiety are gently toxic themselves and enhance the cytotoxicity of topotecan and etoposide at low-toxic concentrations in vitro. Also, novel DCA derivatives were docked at the binding site of the TDP1 and TDP2 enzymes.

## 2. Results and Discussion

### 2.1. Chemistry

**Scheme 1:**
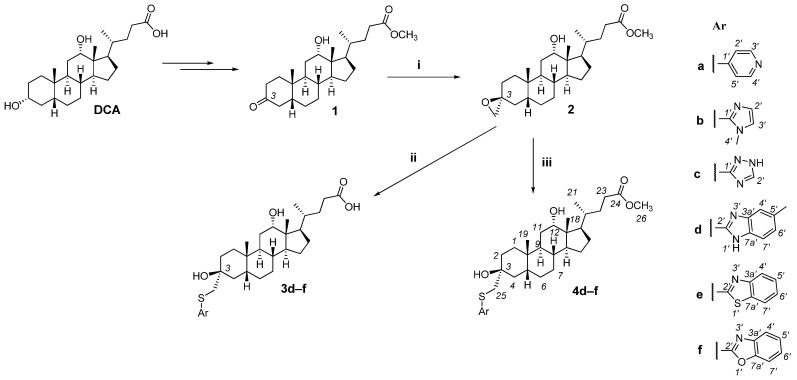
Synthesis of the target DCA derivatives (**3d**–**f**, **4a**–**e**): reagent and conditions: (i) (CH_3_)_3_SOI, NaOMe, DMSO, RT; (ii) (1) ArSH, NaOMe, MeOH, RT; (2) KOH, MeOH-H_2_O, (0° C→RT); and (iii) ArSH, NaOMe, MeOH, RT.

Herein, to study the influence of various heteroaryl functional groups, we synthesized a set of deoxycholic acid derivatives containing a C-3 β-hydroxy group and an α-heteroaryl group in the steroid core (Scheme 1). In the first stage, we synthesized the 3β-oxaspiro derivative of deoxycholic acid **2**, a key intermediate for the synthesis of target compounds. Compound **2** was synthesized according to the literature procedure [23] with a yield of ~40% based on the starting DCA. The epoxy ring formation was carried out via the reaction of the 3-oxo group of the DCA derivative (**1**) with dimethylsulfoxonium methylide formed in situ from trimethylsulfoxonium iodide via the action of a base in DMSO (Corey–Chaykovsky reaction) (Scheme 1).

Next, we proceeded to synthesize the target compounds. As aromatic thiols, we chose 4-mercaptopyridine, five-membered nitrogen-containing heterocycles (2-mercapto-1-methylimidazole and *1H*-1,2,4-triazole-3-thiol), as well as 5-methyl-benzo[*d*]imidazole-2-thiol, 2-mercaptobenzoxazole, and 2-mercaptobenzothiazole. Reactions between **2** and the thiols were carried out in MeOH in the presence of NaOMe. These transformations proceeded under room temperature for about one day, and in each case, a single product was formed (as monitored by TLC and NMR). The ester group was converted to a carboxyl group by alkaline hydrolysis in aqueous methanol. To simplify the processing procedure, we carried out this reaction in *one pot*: after completion of the nucleophilic substitution reaction (TLC control), a mixture of KOH and water (final methanol–water ratio 2:1) was added to the reaction mixture and was then stirred for 3 h at room temperature (TLC control) and processed according to the standard procedure. This modification of the method proved to be successful for all the thiols used, and compounds **3a**–**e** were obtained with yields of 20–63% after purification via flash column chromatography.

Preliminary studies on the inhibition of TDP1 and TDP2 showed a high potential of derivatives **3d**–**3e**, so we also synthesized methyl esters **4d**–**f** with ~65% yield after purification via flash column chromatography.

### 2.2. Biology

#### 2.2.1. Inhibition of Recombinant Enzymes TDP1 and TDP2

To detect TDP1 activity in real time, we used an oligonucleotide biosensor based on the ability of TDP1 to remove fluorophore quenchers from the 3’ end of DNA [29]. The hexadecamer oligonucleotide carried 5(6)-carboxyfluorescein (FAM) at the 5’ end as a fluorophore and Black Hole Quencher-1 (BHQ1) at the 3’ end as a fluorophore quencher. TDP1 inhibitors prevent the removal of fluorophore quenchers, resulting in a decrease in fluorescence intensity. The obtained IC_50_ values of compounds **3a**–**e** and **4d**–**f** are summarized in Table 1. Methyl esters **4d**–**f** suppressed the TDP1 activity at concentrations comparable to that of the reference compound furamidine [30]. The DCA derivatives with free carboxylic group **3a**–**e** demonstrated inhibitory activity in a concentration range of IC_50_ = 14.32–30.2 μM. Overall, compounds **4d**–**e** containing methyl esters were ~25× more active than their corresponding derivatives **3d**–**e** containing free carboxyl group. 

We investigated the activity against the TDP2 enzyme of compounds **3d**–**f** and **4a**–**e** at 500 μM (Table 1). The basis for testing TDP 2 was its ability to remove a tyrosine residue from the 5’ end of an oligonucleotide substrate. The TDP2 reaction products were separated in a polyacrylamide gel (PAGE) with formamide denaturation. It was shown that the compounds with 5-methyl-benzo[*d*]imidazole function at C-3 inhibited the TDP2 enzyme with residual activity of ~25% for compound **3d** and ~14% for compound **4d** (Table 1). Whereas compounds modified with a benzothiadiazole group at C-3 inhibit the TDP2 enzyme with a residual activity of ~2% for compound **3e** and ~49% for compound **4e** (Table 1). Previous DCA-based TDP2 inhibitors containing a 3α-benzyloxy group at C-3 suppressed the activity of this enzyme by no more than 40% (at 1 mM) [20]. Thus, the C-3 β-hydroxyl group, as well as the α-benzene-fused azole ring containing moiety, leads to a significant increase in the effectiveness of TDP2 inhibition. For the most effective TDP2 inhibitors **3d**, **3e**, and **4d**, IC_50_ values were determined, which were found to be in the range of 76–250 μM.

Note that different assay formats were used for studying TDP1 and TDP2. Thus, the value of these assays cannot be directly compared (final enzyme concentrations in these assays were 1.5 nM for TDP1 and 200 nM for TDP2). 

To study the combined action of the compounds in the presence of known antitumor drugs, four compounds, **3d**, **3e**, **4d** and **4e**, were selected that effectively suppress the activity of TDP1 and TDP2.

#### 2.2.2. Cytotoxic/Antiproliferative Action of Combination with Topotecan and Etoposide 

**Figure 2:**
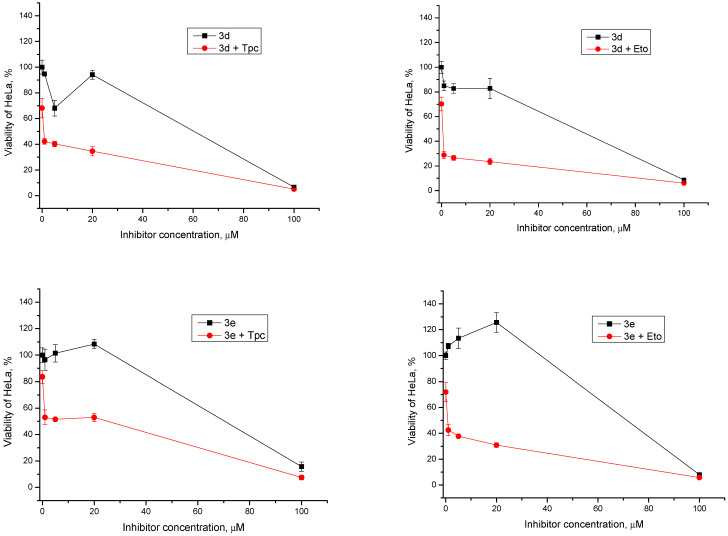

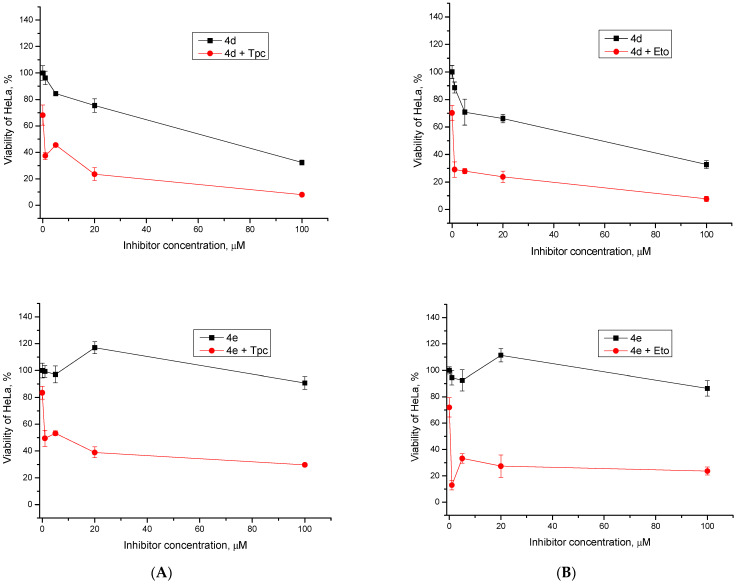
The combined action of compounds **3d**–**e** and **4d**–**e** with 0.5 μM topotecan (Tpc, (**A**)) and 5 µM etoposide (Eto, (**B**)) on the survival of HeLa cells. The error bars show standard deviations.

Topotecan (TOP1 inhibitor) and etoposide (TOP2 inhibitor) were used to study the effect combined with DCA derivatives on the metabolic activity of cells. The HeLa cancer cell line with derivatives **3d**, **3e**, **4d**, and **4e** were used. The results of the MTT assay demonstrated that the derivatives were gently toxic to HeLa cells (Table 1). Only compound **4d** had moderate cytotoxicity (50 µM), while compounds **3d**–**e** and **4e** have CC_50_ values > 75 µM. Topotecan (0.5 µM) and etoposide (5 µM) were used at a concentration with low toxicity (>70% of living cells), selected based on preliminary experiments. In all cases, a significant reduction in cell survival under the influence of drug combinations was seen for all the derivatives (Figure 2). To our surprise, compound **4e**, which was the least effective TDP2 inhibitor, produced the most noticeable combined cytotoxic effect with both topotecan and etoposide. This is probably due to good permeability of the ester inside the cell, where the ester group could be partly hydrolyzed giving free acid which is good inhibitor of TDP2.

### 2.3. Molecular Modeling

The target DCA derivatives **3a**–**e** and **4d**–**f** were docked into the binding sites of the TDP1 (PDB ID: 6W7K, resolution 1.70 Å) [31] and TDP2 (PDB ID: 5J3S, resolution 3.40 Å) [32] enzymes. We used the scoring functions GoldScore (GS) [33], ChemScore (CS) [34,35], ChemPLP (Piecewise Linear Potential) [36], and ASP (Astex Statistical Potential) [37] in the GOLD (v2020.2.0) docking algorithm [38,39].

The binding scores for the TDP1 catalytic pocket are given in Table S1. Although all the ligands have reasonable values, only weak trends were seen when the scores of the active ligands were correlated against their IC_50_ values for ASP (R^2^—0.198), ChemPLP (R^2^—0.235), and GS (R^2^—0.069). However, for CS, a relatively good correlation with R^2^—0.621 was observed (see Figure S1). The correlations between docking scores and IC_50_ values were calculated for TDP2 with the docking scores presented in Table S2. Modest to strong trends were observed for ASP (R^2^—0.454), ChemPLP (R^2^—0.915), CS (R^2^—0.508), and GS (R^2^—0.809). It is important to note that only three compounds have IC_50_ values, so the R^2^ values calculated for TDP2 are unreliable at this point.

**Figure 3:**
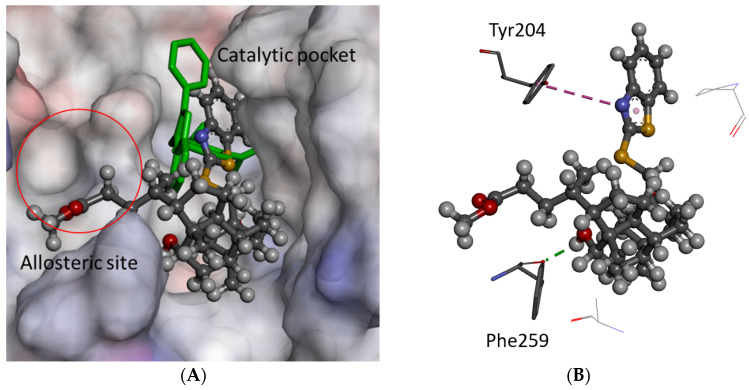
The docked pose of **4e** in the catalytic site of TDP1 as predicted by the ChemPLP scoring function. (**A**) The ligand is shown in the ball-and-stick format. The catalytic pocket is to the right-hand side; the co-crystallized ligand is depicted as green sticks. The ester is predicted to dock into the allosteric site, although no specific interactions are formed (see circled area). The protein surface is rendered; blue depicts regions with a partial positive charge on the surface, red depicts regions with a partial negative charge, and grey shows neutral areas. (**B**) The predicted configuration is depicted as balls-and-sticks, and H-boding is seen between the 12α-hydroxyl group of the DCA steroid backbone and the side chain of Phe259 (stick format). The benzothiazole ring forms a π-stack interaction with Tyr204. The adjacent amino acids (<5 Å), buttressing the ligand, are shown as lines. The amino acids’ hydrogens are not shown for clarity.

The predicted binding poses of **3a**–**e** and **4d**–**f** were investigated. Note that no dominant binding poses were seen by the four scoring functions for both TDP1 and TDP2. Nevertheless, all the ligands occupied the catalytic pocket containing the His263 and His493 amino acid residues in TDP1 and overlapped with the co-crystallized structure. The ChemPLP predicted binding mode of the most active ligand **4e** to TDP1 is shown in Figure 3. The benzothiazole ring moiety overlaps the co-crystallized ligand, whereas the DCA core is in a groove below the co-crystallized ligand. A π-stacked interaction was predicted to form between the benzothiazole ring and Tyr204; furthermore, the native 12α-hydroxy group of the DCA scaffold is predicted to form a H-bond with Phe259 (see Figure 3B). 

Previously published molecular dynamics work suggested that the TDP1 inhibitors occupy an allosteric binding pocket next to the catalytic site, as shown in Figure 3A [40]. Molecular modeling and structural activity relations studies of usnic acid support this idea of enhanced efficacy due to binding to the allosteric site, and the ester moiety of **4e** is indeed docked to this site, potentially explaining its potency. However, this was only seen for ChemPLPand and not the other scoring functions with the same pattern for the other ligands.

**Figure 4:**
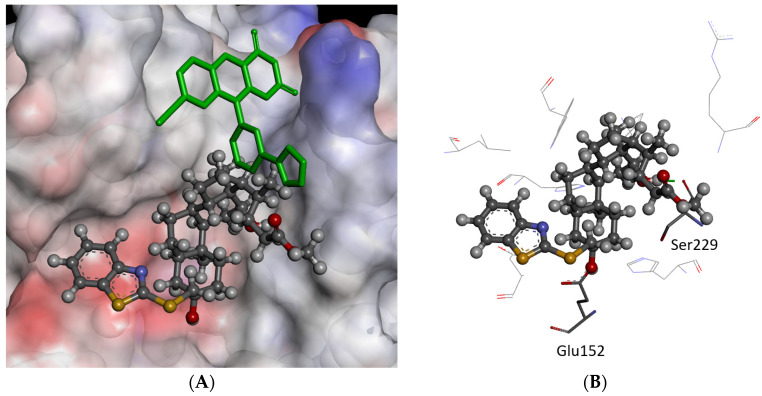
The docked pose of **4e** in the catalytic site of TDP2 as predicted by the ChemPLP scoring function. (**A**) The ligand is shown in the ball-and-stick format. The co-crystallized ligand is depicted as green sticks. The protein surface is rendered; blue depicts regions with a partial positive charge on the surface, red depicts regions with a partial negative charge, and grey shows neutral areas. (**B**) The predicted configuration is depicted as balls-and-sticks, and H-boding is seen between the 12α-hydroxy group of the DCA steroid backbone and the side chain of Ser229 (stick format). Another H-bond interaction is formed between the 3β-hydroxyl group of the DCA steroid backbone and the side chain of Glu152. The adjacent amino acids (<5 Å), buttressing the ligand, are shown as lines. The amino acids’ hydrogens are not shown for clarity.

Compound **4e** was also docked into the binding site of TDP2, as shown in Figure 4. The compound was not predicted to overlap the co-crystallized ligand to any extent. Both hydroxyl groups of the DCA scaffold (native 12α and synthetic 3β) are predicted to form H-bonds with Glu152 and Ser229 (see Figure 4B). 

### 2.4. Prediction of Physicochemical and Drug-like Properties

We calculated molecular descriptors, including MW (molecular weight), log *P* (water–octanol partition coefficient), HD (hydrogen bond donors), HA (hydrogen bond acceptors), PSA (polar surface area), and RB (rotatable bonds) (Table S3). Note that the values of the molecular descriptors lie within lead- and drug-like chemical spaces only for HD, and in the drug-like space for RB, HA, and PSA; MW is only in a known drug space (KDS), and finally, log *P* spans drug-like, KDS, and beyond, i.e., very high values (for the definition of lead-like, drug-like, and KDS regions, see Table S4). Strong correlations were found between the TDP1 IC_50_ values of the ligands and their descriptors for MW (R^2^—0.686), HD (R^2^—0.602), Log *P* (R^2^—0.685), and PSA (R^2^—0.550) favoring large less water-soluble ligands. This means that the activity of the ligands is predominately governed by solvent entropy effects, i.e., the less soluble ligands are pushed into the catalytic site by the water environment. This observation explains the difficulty of the docking algorithm to predict a dominant binding mode for these ligands as their affinity is predominantly governed by solvent effects but not specific binding modes within the catalytic site. Using the same argument of water solubility vs. lipophilicity, the marked activity difference between series **3**, having a carboxyl acid, which is predominantly deprotonated in water and therefore water soluble, and series **4** are the ester derivatives of the acids are thus more lipophilic can be explained. Similar behavior has been seen previously for deoxycholic acid-based derivatives [21], where a strong correlation was seen between activity and the MW and RB molecular descriptors. 

The Known Drug Indexes (KDIs) for the ligands were calculated to gauge the balance of the molecular descriptors (MW, log *P*, HD, HA, PSA, and RB). This method is based on the analysis of drugs in clinical use, i.e., the statistical distribution of each descriptor is fitted to a Gaussian function and normalized to 1, resulting in a weighted index. Both the summation of the indexes (KDI_2a_) and multiplication (KDI_2b_) methods were used [41], as shown for KDI_2a_ in Equation (1) and for KDI_2b_ in Equation (2); the numerical results are given in Table S3.

KDI_2a_ = I_MW_ + I_log P_ + I_HD_+ I_HA_ + I_RB_ + I_PSA_ (1)

KDI_2b_ = I_MW_ × I_log P_ × I_HD_× I_HA_ × I_RB_ × I_PSA_ (2)

The KDI_2a_ values for the ligands range from 3.24 to 4.12 with a theoretical maximum of 6 and an average of 4.08 (±1.27) for known drugs, i.e., relatively small values. The KDI_2b_ range is from 0.01 to 0.07, with a theoretical maximum of 1 and with a KDS average of 0.18 (±0.20). The low numbers for both indexes are due to the large MW and log *P* of the ligands.

### 2.5. Solubility Evaluation of Compounds (***3a***–***e*** and ***4d***–***f***) and in Aqueous Media

**Figure 5:**
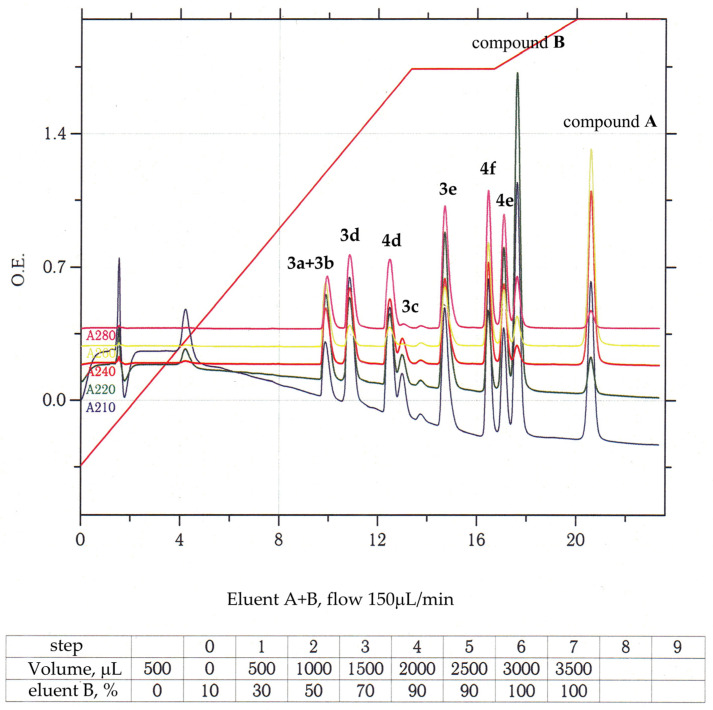
HPLC analyses were carried out on a MilichromA-02 (EcoNova, Novosibirsk, Russia), using a ProntoSIL 120-5-C18 AQ column (BISCHOFF, 2.0 × 75 mm column, grain size 5.0 µm). The mobile phase was Millipore purified water with 0.1% trifluoroacetic acid at a flow rate of 150 µL/min at 35 °C with UV detection at 210, 220, 240, 260, and 280 nm. A typical run time was 25 min with a gradient of 30–100% acetonitrile.

To evaluate the solubility of the obtained DCA derivatives (**3a**–**e**, **4d**–**f**) and 3α,12α-*bis*-methoxy deoxycholic *para*-bromoanilide (compound **A** in Figure 1) and 3α-benzyloxy deoxycholic tryptamide (compound **B** in Figure 1) in aqueous media, we carried out an HPLC experiment on a ProntoSIL-120-5-C18 AQ column, the sorbent of which is silica gel modified with C-18 carbohydrate fragments, using a 0.1% aqueous solution of trifluoroacetic acid (eluent A) with a gradient acetonitrile (eluent B) from 30 to 100% (Figure 5). Based on the nature of reverse-phase chromatography, more hydrophilic compounds appear earlier in the chromatogram than lipophilic structural analogs.

We prepared a mixture of all the compounds in comparable quantities (the chromatography for individual compounds is presented in the Supplementary Material), which was analyzed. As can be seen from the data presented in Figure 5, all new target compounds are more hydrophilic compared to previously obtained derivatives.

## 3. Materials and Methods

### 3.1. Chemistry and General Experimental Procedures

The ^1^H and ^13^C NMR spectra of all new compounds, indicating the numbering of carbon atoms, are given in the Supplementary Materials. The spectra were measured on Bruker spectrometers (Bruker Corporation, Karlsruhe, Germany) as follows: AV-600 (operating frequency 600.30 MHz for ^1^H and 150.95 MHz for ^13^C), DRX-500 (500.13 MHz for ^1^H and 125.76 MHz for ^13^C), AV-400 (400.13 MHz for ^1^H and 100.61 MHz for ^13^C), and AV-300 (300.13 MHz for ^1^H and 75.47 MHz for ^13^C). Solutions of each compound were prepared in CDCl_3_ or in a mixture of CDCl_3_ + CD_3_OD. Chemical shifts were recorded in δ (ppm) using δ 7.24 (^1^H NMR) and δ 76.90 (^13^C NMR) of CHCl_3_ as internal standards. Chemical shift measurements were given in ppm and the coupling constants (J) in hertz (Hz). The hydrogen or carbon atom assignments marked with the same symbols *, # are involved in the exchange of hydrogen of the imidazole or triazole rings and therefore require special attention. Melting points were determined using a METTLER TOLEDO FP900 thermosystem and were uncorrected. Elemental analyses were determined using a EURO EA3000 (Carlo Erba Alanyzers, Milano, Italy) automated CHNS-analyzer. Analyses indicated by the symbols of the elements were within ±0.4% of theoretical values. Optical rotations were measured using a PolAAr 3005 polarimeter (Optical Activity Ltd., Huntingdon, UK). The purity of the final compounds and intermediates for biological testing was >98%, as determined via HPLC analysis. HPLC analyses were carried out on a MilichromA-02 (EcoNova, Novosibirsk, Russia) using a ProntoSIL 120-5-C18 AQ column (BISCHOFF, 2.0 × 75 mm column, grain size 5.0 µm). The mobile phase was Millipore purified water with 0.1% trifluoroacetic acid with acetonitrile (*v*/*v* 25:75) at a flow rate of 150 µL/min at 35 °C with UV detection at 210, 220, 240, 260, and 280 nm; a typical run time was 16 min. The course of all reactions was monitored via TLC analysis using Merck 60 F254 silica gel on aluminum sheets with the eluent CHCl_3_; CHCl_3_–MeOH (25:1.5). Flash column chromatography was performed with silica gel (60–200 µm; Macherey-Nagel Gmb&Co. KG, Duren, Germany). 

### 3.2. Reagents

Deoxycholic acid (99%) and 4-mercaptopyridine (90%) were purchased from abcr GmbH & Co. KG. 2-Mercapto-1-methylimidazole (98%) was purchased from Alfa Aesar. 1*H*-1,2,4-Triazole-3-thiol (97%) and 5-methyl-1*H*-benzo[*d*]imidazole-2-thiol were purchased from Maybrige. 2-Mercaptobenzoxazole (99%) and 2-mercaptobenzothiazole (98%) were purchased from ACROS Organics. All solvents used in the reactions were purified and dried according to previously reported procedures.

Methyl (3β-oxirane)-12α-hydroxy-5β-cholan-24-oate **2** was synthesized according to the literature procedure [23] with a yield of ~40% based on the starting DCA.

#### 3.2.1. General Procedure A for Epoxide Ring Opening with S-Nucleophiles 

A mixture of RSH (1.5 equiv.) and NaOMe (1.5 equiv.) in methanol (10 mL) was stirred for 30 min, and then epoxide **2** (1 equiv.) was added. Then, the reaction mixture was stirred at r. t. for ~5 h until epoxide **2** was completely reacted (monitored by TLC). A solution of KOH (7 equiv.) in water was added to the reaction mixture (the final methanol–water ratio was 2:1), and the mixture was stirred overnight at r. t. The reaction mixture was quenched via the slow addition of NH_4_Cl (sat.), concentrated under reduced pressure, diluted with mixture CH_2_Cl_2_/Et_2_O (1:3 *v*/*v*), and poured on H_2_O. The aqueous layer was extracted using CH_2_Cl_2_/Et_2_O (1:3 *v*/*v*, 3 × 30 mL). The combined organic layers were washed with aqueous NH_4_Cl (sat.) and brined and dried over calcined MgSO_4_. 

#### 3.2.2. General Procedure B for Epoxide Ring Opening with S-Nucleophiles 

A mixture of RSH (1.5 equiv.) and NaOMe (1.5 equiv.) in methanol (10 mL) was stirred for 30 min, and then epoxide **2** (1 equiv.) was added. The reaction mixture was stirred at r. t. for ~5 h until epoxide **2** was completely reacted (monitored by TLC). The reaction mixture was concentrated under reduced pressure, diluted with mixture CH_2_Cl_2_/Et_2_O (1:3 *v*/*v*), and poured on aqueous NH_4_Cl (sat.). The aqueous layer was then extracted with CH_2_Cl_2_/Et_2_O (1:3 *v*/*v*, 3 × 30 mL). The combined organic layers were washed with brine and dried over calcined MgSO_4_.

#### 3.2.3. 3β,12α-Dihydroxy-3α-((pyrydin-4-ylthio)methyl)-5β-cholan-24-oic Acid (**3a**)

The crude product (190 mg) was obtained from epoxide **2** (209 mg, 0.5 mmol), 4-mercaptopyridine (83 mg, 0.75 mmol), and NaOMe (41 mg, 0.75 mmol) in methanol (10 mL) according to the general procedure A. The crude product was purified via flash column chromatography (silica gel, 0.5–5% MeOH in CH_2_Cl_2_) to yield compound **3a** (57 mg, 22%) as a white solid. M.p. 119.6 °C [decomposition]. [$\alpha_{D}^{24.6}$] +32 (*c* 0.39 g/100 mL; CHCl_3_). Anal. Calcd. For C_30_H_45_NO_4_S: C, 69.86; H, 8.79; N, 2.72; O, 12.41; S, 6.22; found C, 69.96; H, 8.89; N, 2.70; S, 6.20. ^1^H NMR (CDCl_3_ + CD_3_OD), 300 MHz): δ = 8.28 (d, 2H, *J*_2′,3′(5′,6′)_ = 6.2, H-3′ and H-5′) and 7.20 (d, 2H, *J*_2′,3′(5′,6′)_ = 1.3, H-4′ and H-5′)—*AB system*, 3.95 (s, 1H, H-12(β)), 3.07 (s, 2H, CH_2_-25), 2.74 (br.s. 3OH + CD_3_OH), 2.31 (m, 1H, CH_2_-23), 2.19 (m, 1H, CH_2_-23′), 0.75–2.0 [m, 30H [0.92 (d, 3H, *J*_21,20_ = 6.2, CH_3_-21), 0.90 (s, 3H, CH_3_-18)], 0.64 (s, 3H, CH_3_-19). ^13^C NMR (CDCl_3_ + CD_3_OD, 75 MHz): δ = 177.11 (s, C-24), 150.54 (s, C-1′), 148.17 (d, C-3′ and C-5′), 121.21 (d, C-2′ and C-5′), 73.00 (d, C-12), 71.76 (s, C-3), 48.13 (d, C-14), 47.11 (d, C-17), 46.34 (t, C-13), 44.96 (t, C-25), 38.00 (d, C-5), 37.15 (t, C-4), 35.60 (d, C-8), 34.97 (d, C-20), 34.15 (s, C-10), 32.68 (d, C-9), 31.25, 30.88, 30.71, 29.51, 28.62 (t, C-11), 27.30 (t, C-16), 26.31 (t, C-6), 25.83 (t, C-7), 23.43 (t, C-15), 23.08 (q, C-19), 17.11 (q, C-21), 12.55 (q, C-18).

#### 3.2.4. 3β,12α-Dihydroxy-3α-((1-methyl-1H-imidazol-2-ylthio)methyl)-5β-cholan-24-oic Acid (**3b**)

The crude product (280 mg) was obtained from epoxide **2** (209 mg, 0.5 mmol), 2-mercapto-1-methylimidazole (86 mg, 0.75 mmol), and NaOMe (41 mg, 0.75 mmol) in methanol (10 mL) according to the general procedure A. The crude product was purified via flash column chromatography (silica gel, 0.5–5% MeOH in CH_2_Cl_2_) to yield compound **3b** (130 mg, 50%) as a white solid. M.p. 119.9 °C [decomposition]. [$\alpha_{D}^{25.5}$] +90 (*c* 0.10 g/100 mL; MeOH). Anal. Calcd. For C_29_H_46_N_2_O_4_S: C, 67.14; H, 8.94; N, 5.40; O, 12.34; S, 6.18; found C, 67.24; H, 8.98; N, 5.45; S, 6.20. ^1^H NMR (CDCl_3_, 300 MHz): δ = 6.92 (d, 1H, *J*_2′,3′_ = 1.3, H-2′) and 6.82 (d, 1H, *J*_2′,3′_ = 1.3, H-3′)—*AB system*, 5.0 (br.s, 3OH), 3.95 (s, 1H, H-12(β)), 3.58 (s, 3H, CH_3_-4′), 3.15 (2H, CH_2_-25—*AB system*), 2.38 (m, 1H, CH_2_-23), 2.22 (m, 1H, CH_2_-23′), 0.9-2.0 [m, 30H [0.96 (d, 3H, *J*_21,20_ = 6.2, CH_3_-21), 0.92 (s, 3H, CH_3_-18)], 0.66 (s, 3H, CH_3_-19). ^13^C NMR (CDCl_3_, 75 MHz): δ = 178.38 (s, C-24), 143.06 (s, C-1′), 127.73 (d, C-2′), 121.81 (d, C-3′), 73.25 (d, C-12), 71.28 (s, C-3), 48.25 (d, C-14), 47.29 (d, C-17), 46.79 (t, C-25), 46.46 (t, C-13), 38.03 (d, C-5), 37.60 (t, C-4), 35.72 (d, C-8), 35.17 (d, C-20), 34.25 (s, C-10), 33.35 (q, C-4′), 32.62 (d, C-9), 31.58, 31.43, 31.18, 30.72, 28.65 (t, C-11), 27.39 (t, C-16), 26.53 (t, C-6), 25.98 (t, C-7), 23.57 (t, C-15), 23.07 (q, C-19), 17.18 (q, C-21), 12.63 (q, C-18).

#### 3.2.5. 3β,12α-Dihydroxy-3α-((1H-1,2,4-triazol-5-ylthio)methyl)-5β-cholan-24-oic Acid (**3c**)

The crude product (280 mg) was obtained from epoxide **2** (209 mg, 0.5 mmol), 1*H*-1,2,4-triazole-3-thiol (76 mg, 0.75 mmol), and NaOMe (41 mg, 0.75 mmol) in methanol (10 mL) according to the general procedure A. The crude product was purified via flash column chromatography (silica gel, 0.5–10% MeOH in CH_2_Cl_2_) to yield compound **3c** (100 mg, 40%) as a white solid. M.p. 95.1–98.4 °C. [$\alpha_{D}^{25.5}$] +46 (*c* 0.56 g/100 mL; MeOH). Anal. Calcd. For C_27_H_43_N_3_O_4_S: C, 64.13; H, 8.57; N, 8.31; O, 12.65; S, 6.34; found C, 64.24; H, 8.63; N, 8.50; S, 6.30. ^1^H NMR (CDCl_3_ + CD_3_OD, 300 MHz): δ = 7.95 (s, 1H, H-2′), 3.91 (s, 1H, H-12(β)), 3.50 (br.s, 3OH), 3.15 (s, 2H, CH_2_-25), 2.30 (m, 1H, CH_2_-23), 2.16 (m, 1H, CH_2_-23′), 0.9-2.0 [m, 30H [0.88 (d, 3H, *J*_21,20_ = 6.2, CH_3_-21), 0.83 (s, 3H, CH_3_-18)], 0.61 (s, 3H, CH_3_-19). ^13^C NMR (CDCl_3_ + CD_3_OD, 75 MHz): δ = 178.38 (s, C-24), 156.89 (s, C-1′), 147.30 (d, C-2′), 73.09 (d, C-12), 71.81 (s, C-3), 48.13 (d, C-14), 46.90 (d, C-17), 46.28 (t, C-13), 45.75 (t, C-25), 37.87 (d, C-5), 36.71 (t, C-4), 35.50 (d, C-8), 35.01 (d, C-20), 34.06 (s, C-10), 32.53 (d, C-9), 31.21, 30.80, 30.67, 30.47, 28.47 (t, C-11), 27.28 (t, C-16), 26.27 (t, C-6), 25.81 (t, C-7), 23.44 (t, C-15), 22.89 (q, C-19), 16.96 (q, C-21), 12.49 (q, C-18).

#### 3.2.6. 3β,12α-Dihydroxy-3α-(5-methyl-1H-benzo[d]imidazol-2-ylthio)methyl)-5β-cholan-24-oic Acid (**3d**)

The crude product (304 mg) was obtained from epoxide **2** (209 mg, 0.5 mmol), 5-methyl-1H-benzo[d]imidazole-2-thiol (113 mg, 0.75 mmol), and NaOMe (21 mg, 0.38 mmol) in methanol (10 mL) according to the general procedure A. The crude product was purified via flash column chromatography (silica gel, 0.5–10% MeOH in CH_2_Cl_2_) to yield compound **3d** (135 mg, 48%) as a white solid. M.p. 188.9 °C [decomposition]. [$\alpha_{D}^{25.5}$] +100 (*c* 0.12 g/100 mL; MeOH). Anal. Calcd. For C_33_H_48_N_2_O_4_S: C, 69.68; H, 8.51; N, 4.92; O, 11.25; S, 5.64; found C, 69.70; H, 8.65; N, 4.85; S, 5.54. ^1^H NMR (CDCl_3_ + CD_3_OD, 500 MHz): δ = 8.86 (d, 1H, *J*_6′,7′_ = 8.0, H-7′), 7.17 (s, 1H, H-3), 6.91 (d, 1H, *J*_6′,7′_ = 8.0, H-6′), 3.96 (s, 1H, H-12(β)), 3.26 (d, 1H, *J* = 14.3, H-25) and 3.31 (d, 1H, *J* = 14.3, H-25′)—*AB system*, 2.25–2.40 (m, 4H, [CH_3_-8′ and H-23]), 2.24 (m, 1H, CH_2_-23′), 0.75–2.00 [m, 30H, [0.93 (d, 3H, *J*_21,20_ = 6.2, CH_3_-21), 0.87 (s, 3H, CH_3_-18)], 0.62 (s, 3H, CH_3_-19). ^13^C NMR (CDCl_3_ + CD_3_OD, 125 MHz): δ = 177.00 (s, C-24), 150.97 (s, C-2′), 133.02 (s, C-5′), 131.66* (s, C-3a′), 130.21* (s, C-7a′), 124.00 (d, C-6′), 110.12^#^ (d, C-4′), 109.52^#^ (d, C-7′), 73.30 (d, C-12), 71.76 (s, C-3), 49.09 (q, C-26). 46.89 (d, C-14), 47.03 (d, C-17), 46.05 (t, C-13), 45.57 (t, C-25), 37.77 (d, C-5), 37.31 (t, C-4), 35.40 (d, C-8), 34.70 (d, C-20), 33.91 (s, C-10), 32.54 (d, C-9), 31.25, 31.26, 30.86, 30.64, 28.42 (t, C-11), 27.34 (t, C-16), 26.31 (t, C-6), 25.77 (t, C-7), 23.47 (t, C-15), 22.90 (q, C-19), 21.22 (q, C-8′) 17.05 (q, C-21), 12.49 (q, C-18).

#### 3.2.7. 3β,12α-Dihydroxy-3α-(benzo[d]thiazol-2-ylthio)methyl)-5β-cholan-24-oic Acid (**3e**)

The crude product (300 mg) was obtained from epoxide **2** (209 mg, 0.5 mmol), 2-mercaptobenzothiazole (125 mg, 0.75 mmol), and NaOMe (41 mg, 0.75 mmol) in methanol (10 mL) according to the general procedure A. The crude product was purified via flash column chromatography (silica gel, 0.5–3% MeOH in CH_2_Cl_2_) to yield compound **3e** (180 mg, 63%) as a white solid. M.p. 106.2 °C [decomposition]. [$\alpha_{D}^{24.5}$] +60 (*c* 0.18 g/100 mL; CHCl_3_). Anal. Calcd. For C_32_H_45_NO_4_S_2_: C, 67.21; H, 7.93; N, 2.45; O, 11.19; S, 11.21; found C, 67.39; H, 8.03; N, 2.40; S, 11.10. ^1^H NMR (CDCl_3_, 400 MHz): δ = 7.80 (m, 1H, *J* = 8.0, H-4′), 7.69 (m, 1H, *J* = 7.7, H-7′), 7.37 (m, 1H, *J* = 7.7, *J* = 1.1, H-5′), 7.26 (m, 1H, *J* = 7.7, *J* = 1.1, H-6′), 4.50 (br.s, 3OH), 3.98 (s, 1H, H-12(β)), 3.50 (s, 2H, CH_2_-25), 2.38 (m, 1H, CH_2_-23), 2.24 (m, 1H, CH_2_-23′), 0.8–2.0 [m, 30H [0.95 (d, 3H, *J*_21,20_ = 6.2, CH_3_-21), 0.94 (s, 3H, CH_3_-18)], 0.67 (s, 3H, CH_3_-19). ^13^C NMR (CDCl_3_, 100 MHz): δ = 179.12 (s, C-24), 168.83 (s, C-2′), 152.15 (s, C-3a′), 135.22 (s, C-7a′), 126.25 (d, C-5′), 124.38 (d, C-6′), 121.08 (d, C-4′), 120.85 (d, C-7′), 73.27 (d, C-12), 71.43 (s, C-3), 48.21 (d, C-14), 47.14 (d, C-17), 47.11 (t, C-13), 46.35 (t, C-25), 38.03 (d, C-5), 37.69 (t, C-4), 35.66 (d, C-8), 34.93 (d, C-20), 34.16 (s, C-10), 32.75 (d, C-9), 31.88, 31.37, 30.86, 30.56, 28.70 (t, C-11), 27.33 (t, C-16), 26.43 (t, C-6), 25.89 (t, C-7), 23.45 (t, C-15), 23.10 (q, C-19), 17.16 (q, C-21), 12.59 (q, C-18).

#### 3.2.8. Methyl 3α-(5-methyl-1H-benzo[d]imidazol-2-ylthio)methyl)-3β,12α-dihydroxy-5β-cholan-24-oate (**4d**)

The crude product (150 mg) was obtained from epoxide **2** (104 mg, 0.25 mmol), 5-methyl-1H-benzo[*d*]imidazole-2-thiol (55 mg, 0.38 mmol), and NaOMe (21 mg, 0.38 mmol) in methanol (10 mL) according to the general procedure B. The crude product was purified via flash column chromatography (silica gel, 0.5–5% MeOH in CH_2_Cl_2_) to yield compound **4d** (72 mg, 50%) as a white solid. M.p. 112.1 °C [decomposition]. [$\alpha_{D}^{25.5}$] +24 (*c* 0.13 g/100 mL; MeOH). Anal. Calcd. For C_34_H_50_N_2_O_4_S: C, 70.06; H, 8.65; N, 4.81; O, 10.98; S, 5.50; found C, 70.15; H, 8.70; N, 4.70; S, 5.50. ^1^H NMR (CDCl_3_, 600 MHz): δ = 7.20 (m, 2H, H-3′ and H-7′), 7.93 (m, 1H, H-6′), 4.00 (s, 1H, H-12(β)), 3.64 (s, 3H, CH_3_-26), 3.18 (s, 2H, CH_2_-25), 2.36 (s, 3H, CH_3_-8′), 2.38 (m, 1H, CH_2_-23), 2.24 (m, 1H, CH_2_-23′), 0.9–2.00 [m, 30H, [0.97 (d, 3H, *J*_21,20_ = 6.2, CH_3_-21), 0.93 (s, 3H, CH_3_-18)], 0.67 (s, 3H, CH_3_-19). ^13^C NMR (CDCl_3_, 125 MHz): δ = 174.33 (s, C-24), 150.80 (s, C-2′), 132.47* (s, C-3a′), 131.66 (s, C-5′), 130.21* (s, C-7a′), 123.20 (d, C-6′), 109.62^#^ (d, C-4′), 106.13^#^ (d, C-7′), 73.09 (d, C-12), 71.21 (s, C-3), 51.10 (q, C-26). 47.93 (d, C-14), 47.03 (d, C-17), 46.05 (t, C-13), 45.57 (t, C-25), 37.77 (d, C-5), 37.31 (t, C-4), 35.40 (d, C-8), 34.70 (d, C-20), 33.91 (s, C-10), 32.51 (d, C-9), 31.44, 31.13, 30.72, 30.47, 28.49 (t, C-11), 27.01 (t, C-16), 26.17 (t, C-6), 25.61 (t, C-7), 23.17 (t, C-15), 22.83 (q, C-19), 21.11 (q, C-8′) 16.95 (q, C-21), 12.31 (q, C-18).

#### 3.2.9. Methyl 3α-(benzo[d]thiazol-2-ylthio)methyl)-3β,12α-dihydroxy-5β-cholan-24-oate (**4e**)

The crude product (150 mg) was obtained from epoxide **2** (104 mg, 0.25 mmol), 2-mercaptobenzothiazole (62 mg, 0.38 mmol), and NaOMe (21 mg, 0.38 mmol) in methanol (10 mL) according to the general procedure B. The crude product was purified via flash column chromatography (silica gel, 0.5–2% MeOH in CH_2_Cl_2_) to yield compound **4e** (97 mg, 66%) as a white solid. M.p. 74.5 °C [decomposition]. [$\alpha_{D}^{24.6}$] +45 (*c* 0.98 g/100 mL; CHCl_3_). Anal. Calcd. For C_33_H_47_NO_4_S_2_: C, 67.65; H, 8.09; N, 2.39; O, 10.92; S, 10.95; found C, 67.70; H, 8.12; N, 2.40; S, 10.80. ^1^H NMR (CDCl_3_, 600 MHz): δ = 7.80 (m, 1H, *J* = 8.0, H-4′), 7.69 (m, 1H, *J* = 7.7, H-7′), 7.37 (m, 1H, *J* = 7.7, *J* = 1.1, H-5′), 7.26 (m, 1H, *J* = 7.7, *J* = 1.1, H-6′), 4.28 (s, OH), 3.97 (s, 1H, H-12(β)), 3.64 (s, 3H, CH_3_-26), 3.44 (s, 2H, CH_2_-25), 2.38 (m, 1H, CH_2_-23), 2.24 (m, 1H, CH_2_-23′), 1.0–1.96 [m, 24H], 0.95 (d, 3H, *J*_21,20_ = 6.2, CH_3_-21), 0.95 (s, 3H, CH_3_-18)], 0.67 (s, 3H, CH_3_-19). ^13^C NMR (CDCl_3_, 125 MHz): δ = 174.52 (s, C-24), 168.52 (s, C-2′), 152.30 (s, C-3a′), 135.35 (s, C-7a′), 126.03 (d, C-5′), 124.37 (d, C-6′), 121.16 (d, C-4′), 120.86 (d, C-7′), 73.14 (d, C-12), 71.35 (s, C-3), 51.34 (q, C-26). 48.31 (d, C-14), 47.32 (d, C-17), 47.21 (t, C-13), 46.44 (t, C-25), 38.12 (d, C-5), 37.86 (t, C-4), 35.78 (d, C-8), 34.95 (d, C-20), 34.21 (s, C-10), 32.88 (d, C-9), 32.07, 31.42, 30.95, 30.82, 28.90 (t, C-11), 28.68 (t, C-16), 27.32 (t, C-6), 26.50 (t, C-7), 23.46 (t, C-15), 23.16 (q, C-19), 17.26 (q, C-21), 12.63 (q, C-18).

#### 3.2.10. Methyl 3α-(benzo[d]oxazol-2-ylthio)methyl)-3β,12α-dihydroxy-5β-cholan-24-oate (**4f**)

The crude product (140 mg) was obtained from epoxide **2** (104 mg, 0.25 mmol), 2-mercaptobenzoxazole (57 mg, 0.38 mmol), and NaOMe (21 mg, 0.38 mmol) in methanol (10 mL) according to the general procedure B. The crude product was purified via flash column chromatography (silica gel, 0.5–2% MeOH in CH_2_Cl_2_) to yield compound **4f** (89 mg, 63%) as a white solid. M.p. 75.0 °C [decomposition]. [$\alpha_{D}^{24.6}$] +46 (*c* 0.15 g/100 mL; CHCl_3_). Anal. Calcd. For C_33_H_47_NO_5_S: C, 69.56; H, 8.31; N, 2.46; O, 14.04; S, 5.63; found C, 69.69; H, 8.40; N, 2.40; S, 5.50. ^1^H NMR (CDCl_3_, 400 MHz): δ = 7.55 (m, 1H, H-4′), 7.43 (m, 1H, H-7′), 7.27 (m, 2H, H-5′ and H-6′), 4.02 (s, 1H, H-12(β)), 3.68 (s, 3H, CH_3_-26), 3.61 (s, OH), 3.47 (s, 2H, CH_2_-25), 2.38 (m, 1H, CH_2_-23), 2.24 (m, 1H, CH_2_-23′), 0.95–1.96 [m, 30H, [0.99 (d, 3H, *J*_21,20_ = 6.2, CH_3_-21), 0.98 (s, 3H, CH_3_-18)], 0.71 (s, 3H, CH_3_-19). ^13^C NMR (CDCl_3_, 100 MHz): δ = 174.52 (s, C-24), 166.52 (s, C-2′), 151.91 (s, C-7a′), 141.17 (s, C-3a′), 124.31 (d, C-5′), 123.95 (d, C-6′), 118.13 (d, C-4′), 109.84 (d, C-7′), 73.13 (d, C-12), 71.64 (s, C-3), 51.40 (q, C-26). 48.28 (d, C-14), 47.30 (d, C-17), 46.44 (t, C-13), 46.77 (t, C-25), 38.09 (d, C-5), 37.51 (t, C-4), 35.76 (d, C-8), 34.98 (d, C-20), 34.22 (s, C-10), 32.85 (d, C-9), 31.77, 31.36, 30.97, 30.82, 28.88 (t, C-11), 27.34 (t, C-16), 26.46 (t, C-6), 25.93 (t, C-7), 23.49 (t, C-15), 23.20 (q, C-19), 17.27 (q, C-21), 12.66 (q, C-18).

### 3.3. Biology 

#### 3.3.1. Detection of TDP1 Activity

TDP1 activity was detected as described in [31]. We measured the fluorescence intensity after quencher cleavage via a fluorophore quencher–coupled DNA oligonucleotide 5eotide after quencher cleavage due to enzyme activity. The reaction mixtures contained TDP1 buffer (50 mM Tris, HCl pH 8.0, 50 mM NaCl, and 7 mM β-mercaptoethanol), 50 nM biosensor, and different concentrations of inhibitors. The reaction was triggered by adding TDP1 to a final concentration of 1.5 nM. The fluorophore quencher–coupled DNA oligonucleotide was synthesized in the Laboratory of Nucleic Acid Chemistry at the Institute of Chemical Biology and Fundamental Medicine (Novosibirsk, Russia).

The reaction mixtures were incubated at room temperature in a 96-well plate, and fluorescence was measured using the POLARstar OPTIMA fluorimeter (BMG LABTECH, GmbH, Ortenberg, Germany) every 55 s during the linear phase (here, data from minute 0 to minute 8). From the data on changes in fluorescence in the presence of inhibitors, the IC_50_ values were determined (the inhibitor concentration at which the enzyme activity is reduced by half) in a minimum of three independent experiments. Data calculation was carried out using the software MARS Data Analysis 2.0 (BMG LABTECH, Ortenberg, Germany).

#### 3.3.2. Gel-Based TDP2 Activity Assay

TDP2 activity was detected as described in [42]. We separated the products of the reaction of cleavage of the tyrosine residue from the 5′-end of the 3e FAM-labeled oligonucleotide in a polyacrylamide gel under denaturing conditions. The oligonucleotide 5ith tyrosine r GTC AGG GTC TTC C-FAM-3G was synthesized at the Novosibirsk State University, Russia, as described [30]. The reaction mixtures contained TDP1 buffer (500 mM Tris-HCl pH 8.0, 40 mM NaCl, 1 mM DTT, 0.05 mg/mL BSA (Sigma-Aldrich, St. Luis, MO, USA)), 100 nM oligonucleotide substrate, and inhibitors. The reaction was triggered by adding TDP2 to a final concentration of 200 nM. 

The reaction mixtures were incubated for 15 min at 37 °C. The reaction products were analyzed using Typhoon FLA 9500 phosphorimager (GE Healthcare, Boston, MA, USA). Each experiment was carried out in three independent replicates. The data were analyzed with QuantityOne 4.6.7 software. 

#### 3.3.3. Study of Cytotoxic/Antiproliferative Effects of Compounds

Analysis of cytotoxic/antiproliferative effects of the compounds **3d**–**e** and **4d**–**f** were examined against cell line HeLa (human cervical cancer) using MTT Cell Proliferation and Cytotoxicity Assay (Dia-m, Moscow, Russia), according to the manufacturer’s protocols. The cells were seeded in 96-well plates in quadruplicates at 3 × 10^3^ cells/well. The cells were grown in Dulbecco’s Modified Eagle—Medium (DMEM) (AppliChem, Darmstadt, Germany) with Antibiotic-Antimycotic (100 IU/mL penicillin, 0.1 μg/mL streptomycin, 0.25 µg/mL amphotericin) (1×) (Gibco, Grand Island, NE, USA) and 10% of fetal bovine serum (Gibco, Grand Island, NE, USA) in a 5% CO_2_ atmosphere at 37 °C. After the formation of a 50% monolayer, tested compounds were added to the medium, and the cell culture was monitored for 72 h at 37 °C. DCA derivatives were dissolved in 100% DMSO and topotecan/etoposide in water. Control cells were grown in the presence of the same volume of DMSO, those cells with the investigated compound. Thereafter, aliquots of [3-(4,5-dimethylthiazol-2-yl)-2,5-diphenyltetrazolium bromide] (MTT) solution (10 μL, 5 mg/mL) were added to each well and the cells were incubated for an additional 2 h. Then, the medium was removed from the wells, and 100 μL of *iso*-propanol was added to dissolve blue formazan crystals formed within live cells; the optical density was measured at test and reference wavelengths (570 nm and 620 nm, respectively) using CLARIOstar fluorimeter (BMG LABTECH, GmbH, Ortenberg, Germany). The experiments were repeated at least three times. 

Next, the ability of the selected compounds to enhance the cytotoxic effect of topotecan and etoposide on the HeLa cell line was studied. CC_50_ values (50% cytotoxic concentration) were also calculated for the test compounds in the presence of topotecan or etoposide at fixed concentrations. For evaluation, three independent MTT assays were performed as described above with test substances in combination with topotecan at a final concentration of 0.5 μM and etoposide at a final concentration of 5 μM per well.

### 3.4. Modeling and Screening

The crystal structure of TDP1 (PDB ID: 6W7K, resolution 1.70 Å) [31] and TDP2 (PDB ID: 5J3S, resolution 3.40Å) [32] enzymes were obtained from the Protein Data Bank (PDB) [43,44]. The GOLD (v2020.2.0) software suite was used to prepare the crystal structures for docking of all the ligands, i.e., hydrogen atoms were added, all the waters were removed and the co-crystallized ligands 4-[(2-phenylimidazo[1,2-*a*]pyridin-3-yl)amino]benzene-1,2-dicarboxylic acid (TG7–TDP1) and 2,4-dioxo-10-[3-(1*H*-tetrazol-5-yl)phenyl]-2,3,4,10-tetrahydropyrimido[4,5-*b*]quinoline-8-carbonitrile (6FQ–TDP2) were found. The Scigress software version FQ 3.4.4 [45] was used to make the compounds, which, in turn, the global minimum and structural optimization were carried out using the MM3 [46,47,48] force field in conjunction with the CONFLEX method [49]. The location of the co-crystallized ligands TG7 and 6FQ was set as the center of the docking (10 Å radius). The default search efficiency (100%) was used with fifty docking rungs per ligand. The basic amino acids lysine and arginine were defined as protonated; also, aspartic and glutamic acids were kept deprotonated. The GoldScore (GS) [33] and ChemScore (CS) [34,35] Piecewise Linear Potential (ChemPLP) [22] and Astex Statistical Potential (ASP) [36,37] scoring functions were used for the binding modes and predicted binding energies of the ligands using the GOLD v2020.2.0 software.

The molecular descriptors were calculated using the QikProp 6.2 [50] software package. The reliability of this package is established [51]. The Known Drug Indexes (KDI) were derived from the molecular descriptors as shown by Eurtivong and Reynisson [29]. For application in Excel, columns for each descriptor were made, and these equations applied derived the KDI numbers for each property as follows: KDI MW: = EXP(−((*MW* − 371.76)^2)/(2*(112.76^2))), KDI Log *P*: = EXP(−((*LogP* − 2.82)^2)/(2*(2.21^2))), KDI HD: = EXP(−((*HD* − 1.88)^2)/(2*(1.7^2))), KDI HA: = EXP(−((*HA* − 5.72)^2)/(2*(2.86^2))), KDI RB: = EXP(−((*RB* − 4.44)^2)/(2*(3.55^2))), and KDI PSA: = EXP(−((*PSA* − 79.4)^2)/(2*(54.16^2))). These equations can be copied into an Excel spreadsheet, and the descriptor name (e.g., *MW*) is substituted with the value in the relevant column. To calculate KDI_2A_ and KDI_2B,_ the following equations were used: = (KDI MW + KDI Log *P* + KDI HD + KDI HA + KDI RB + KDI PSA), KDI_2B_: = (KDI MW × KDI LogP × KDI HD × KDI HA × KDI RB × KDI PSA).

## 4. Conclusions

Deoxycholic acid derivatives with various heterocyclic functional groups at C-3 on the steroid core were synthesized and evaluated as inhibitors of TDP1 and TDP2. **4e** is the most potent ligand for TDP1 (IC_50_—0.63 ± 0.03 µM) and **3e** for TDP2 (IC_50_—76 ± 13 µM). All the ligands had no modes of cytotoxicity against HeLa cancer cells. The methyl esters of DCA derivatives **4d**–**e**, as well as their acid counterparts **3d**–**e** with benzothiazole and benzimidazole moieties, enhanced the cytotoxicity of topotecan and etoposide in low-toxic concentrations in vitro. Molecular modeling studies showed a good fit of the ligands into the respective binding sites of TDP1 and TDP2, but no dominant poses were predicted. The ligand tends to have large MW and log *P* values, and TDP1 activity correlates reasonably with these molecular descriptors as well as HA and PSA. The results here contribute to the quest for potent dual TDP1 and TDP2 inhibitors clinical candidates.

## Figures and Tables

**Table 1:** TDP1 and TDP2 inhibitory activity of compounds **3a**–**e** and **4d**–**f**.

Compound		TDP1 IC_50_ ^1^, μM	TDP2 IC_50_ ^1^, μM	TDP2 ^2^, %	HeLa CC_50_ ^3^, μM
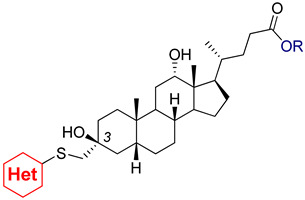					
**3a**	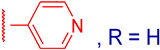	22 ± 2	ND	92 ± 3	ND
**3b**	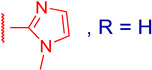	30 ± 7	ND	≥100	ND
**3c**	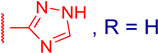	32 ± 4	ND	≥100	ND
**3d**	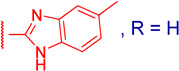	25 ± 4	250 ± 30	24 ± 5	63 ± 6
**3e**	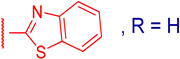	14 ± 2	76 ± 13	2 ± 3	75 ± 13
**4d**	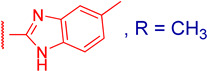	0.94 ± 0.04	218 ± 7	14 ± 5	50 ± 13
**4e**	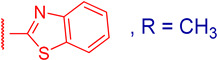	0.63 ± 0.03	ND	49 ± 2	>100
**4f**	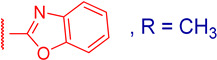	1.8 ± 0.5	ND	≥100	ND
	Fur ^4^	1.2 ± 0.3	ND	ND	ND
Topotecan		ND	ND	ND	0.9 ± 0.1
Etoposide		ND	ND	ND	6.2 ± 0.6

^1^ IC_50_—concentration that inhibits the activity of an enzyme by 50%. ^2^ The residual activity of TDP2 (%) at 500 µM for **3a**–**e**–**4d**–**f**. ^3^ CC_50_—concentration causing 50% cell death after 72 h of treatment measured using the colorimetric MTT assay. ^4^ Fur—furamidine, commercial TDP1 inhibitor [30]. ND—not determined.

## Data Availability

The data presented in this study are available on request from the corresponding author.

## Supplementary Materials

The following are available online at https://www.mdpi.com/article/10.3390/molecules29030581/s1, Chemistry: NMR ^1^H and ^13^C of DCA derivatives (**3a**–**e** and **4e**–**f**); Molecular modeling; Table S1. The binding affinities as predicted by the scoring functions used to the catalytic TDP1 binding pocket and their measured IC_50_ values. Figure S1. The correlation plot of measured IC_50_ values against their CS counterparts. Table S2. The binding affinities as predicted by the scoring functions used to the catalytic TDP2 binding pocket and their measured IC_50_ values. Table S3. The molecular descriptors and their corresponding Known Drug Indexes 2a and 2b (KDI_2a/2b_). Table S4. Definition of lead-like, drug-like, and Known Drug Space (KDS) in terms of molecular descriptors. The values given are the maxima for each descriptor for the volumes of chemical space used.
