# Supplementary material for: New Dual Inhibitors of Tyrosyl-DNA Phosphodiesterase 1 and 2 Based on Deoxycholic Acid: Design, Synthesis, Cytotoxicity, and Molecular Modeling
Source: Molecules. 2024 Jan 24;29(3):581. doi: 10.3390/molecules29030581 (PMC10856758; doi:10.3390/molecules29030581)
Supplement: Supplementary file 1 [file molecules-29-00581-s001.zip › molecules-2737291-supplementary.pdf]

New Dual Inhibitors of Tyrosyl-DNA Phosphodiesterase 1 and 2 Based on Deoxycholic Acid: Design, Synthesis, Cytotoxicity, and Molecular Modeling

Oksana V. Salomatina [1], Tatyana E. Kornienko [2], Alexandra L. Zakharenko [2], Nina I. Komarova [1], Chigozie Achara [3], Jóhannes Reynisson [3], Nariman F. Salakhutdinov [1], Olga I. Lavrik [2] and Konstantin P. Volcho [1,*]

[1] N.N. Vorozhtsov Novosibirsk Institute of Organic Chemistry, SB RAS, 9, Lavrent'ev Ave., Novosibirsk 630090, Russia; ana@nioch.nsc.ru (O.V.S.); komar@nioch.nsc.ru (N.I.K.); anvar@nioch.nsc.ru (N.F.S.)

[2] Institute of Chemical Biology and Fundamental Medicine, SB RAS, 8, Lavrent'ev Ave., Novosibirsk 630090, Russia; t.kornienko1995@gmail.com (T.E.K.); sashaz@niboch.nsc.ru (A.L.Z.); lavrik@niboch.nsc.ru (O.I.L.)

[3] School of Pharmacy and Bioengineering, Keele University, Hornbeam Building, Newcastle-under-Lyme, Staffordshire ST5 5BG, UK; a.achara@keele.ac.uk (C.A.); j.reynisson@keele.ac.uk (J.R.)

* Correspondence: volcho@nioch.nsc.ru; Tel.: +7-383-3308-870

NMR ^{1}H and **^{13}C** of DCA derivatives (**2, 3a-e, 4d-f**)

Table S1. The binding affinities as predicted by the scoring functions used to the catalytic TDP1 binding pocket and their measured IC_{50} values.

Figure S1. The correlation plot of measured IC_{50} values against their CS counterparts.

Table S2. The binding affinities as predicted by the scoring functions used to the catalytic TDP2 binding pocket and their measured IC_{50} values.

Table S3. The molecular descriptors and their corresponding Known Drug Indexes 2a and 2b ($KDI_{2a/2b}$).

Table S4. Definition of lead-like, drug-like and Known Drug Space (KDS) in terms of molecular descriptors. The values given are the maxima for each descriptor for the volumes of chemical space used.

HPLC analyses for compound **3a-e, 4d-f** and 3α,12α-*bis*-methoxy deoxycholic *para*-bromoanilide (compound **A**) and 3α-benzyloxy deoxycholic tryptamide (compound **B**)

Spectrum of compound **2,** ^{1}H NMR, 500MHz, $CDCl_3$

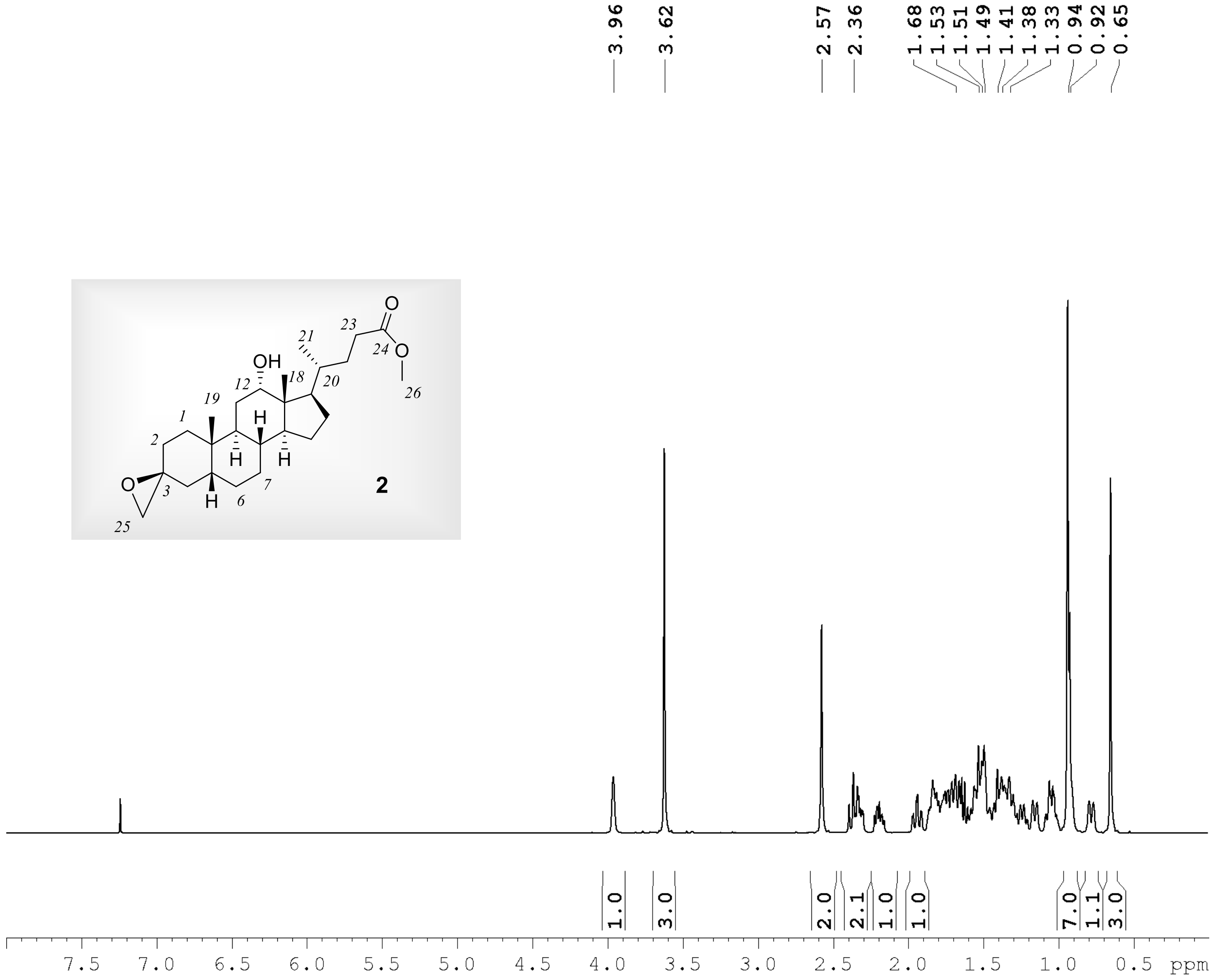

Spectrum of compound **2,** ^{13}C NMR, 125MHz, $CDCl_3$

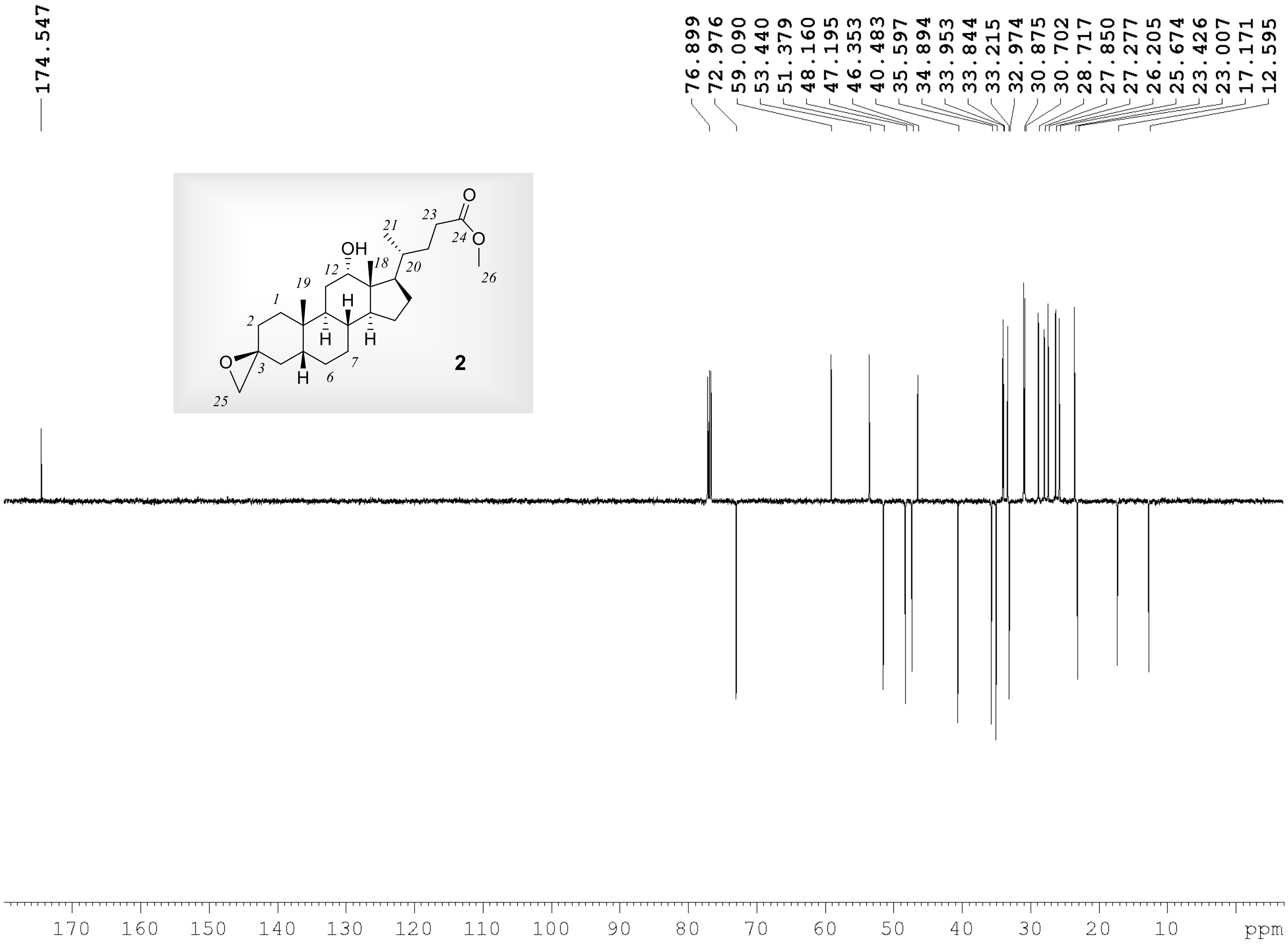
174.547
76.899
72.976
59.090
53.440
51.379
48.160
47.195
46.353
40.483
35.597
34.894
33.953
33.844
33.215
32.974
30.875
30.702
28.717
27.850
27.277
26.205
25.674
23.426
23.007
17.171
12.595
OH
O
1
2
3
6
7
12
18
19
20
21
23
24
25
26
H
2
170 160 150 140 130 120 110 100 90 80 70 60 50 40 30 20 10 ppm

Spectrum of compound **3a**, ^{1}H NMR, 300MHz, $CDCl_3$+CD_3OD

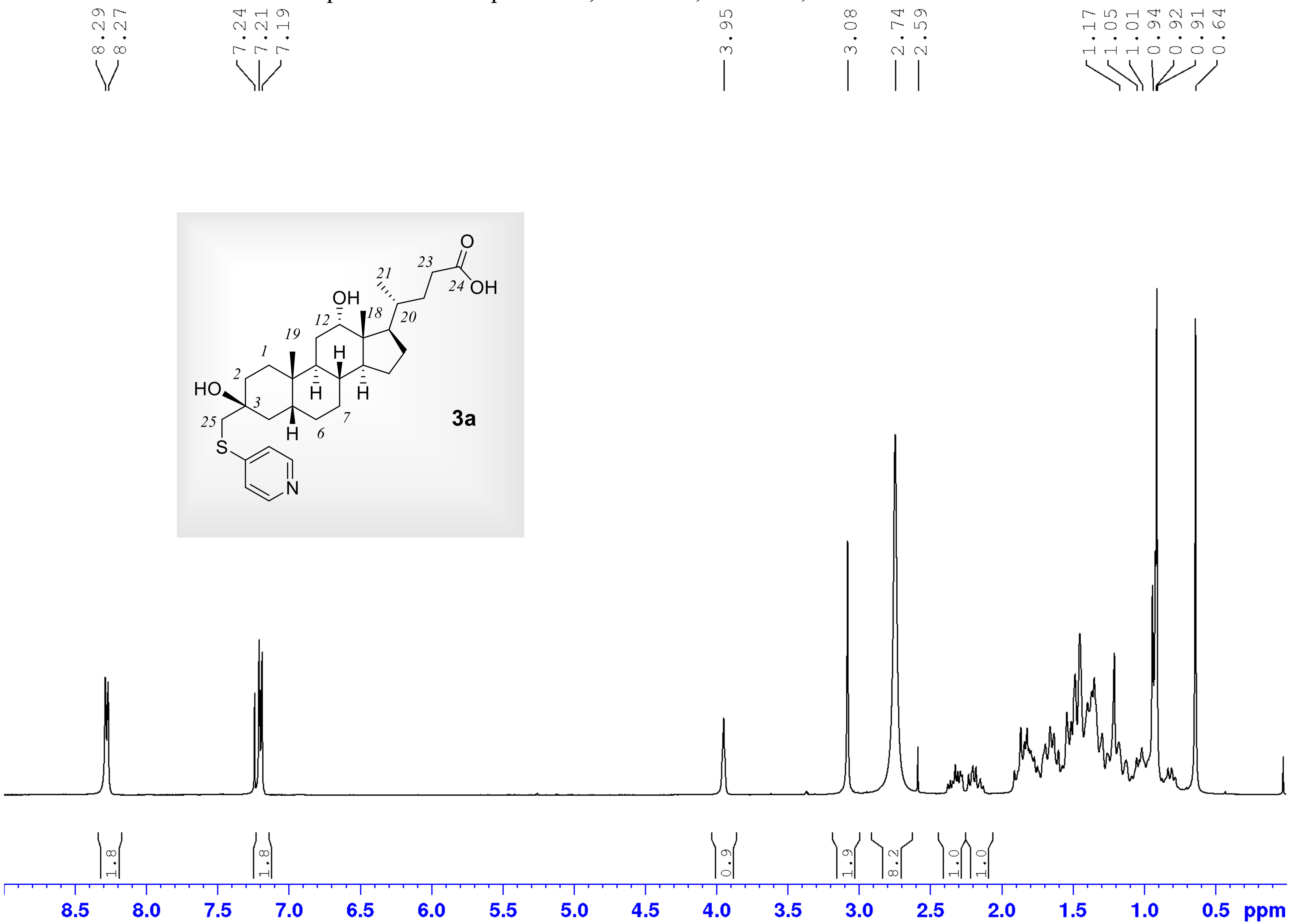

Spectrum of compound **3a**, ^{13}C NMR, 75MHz, $CDCl_3$+CD_3OD

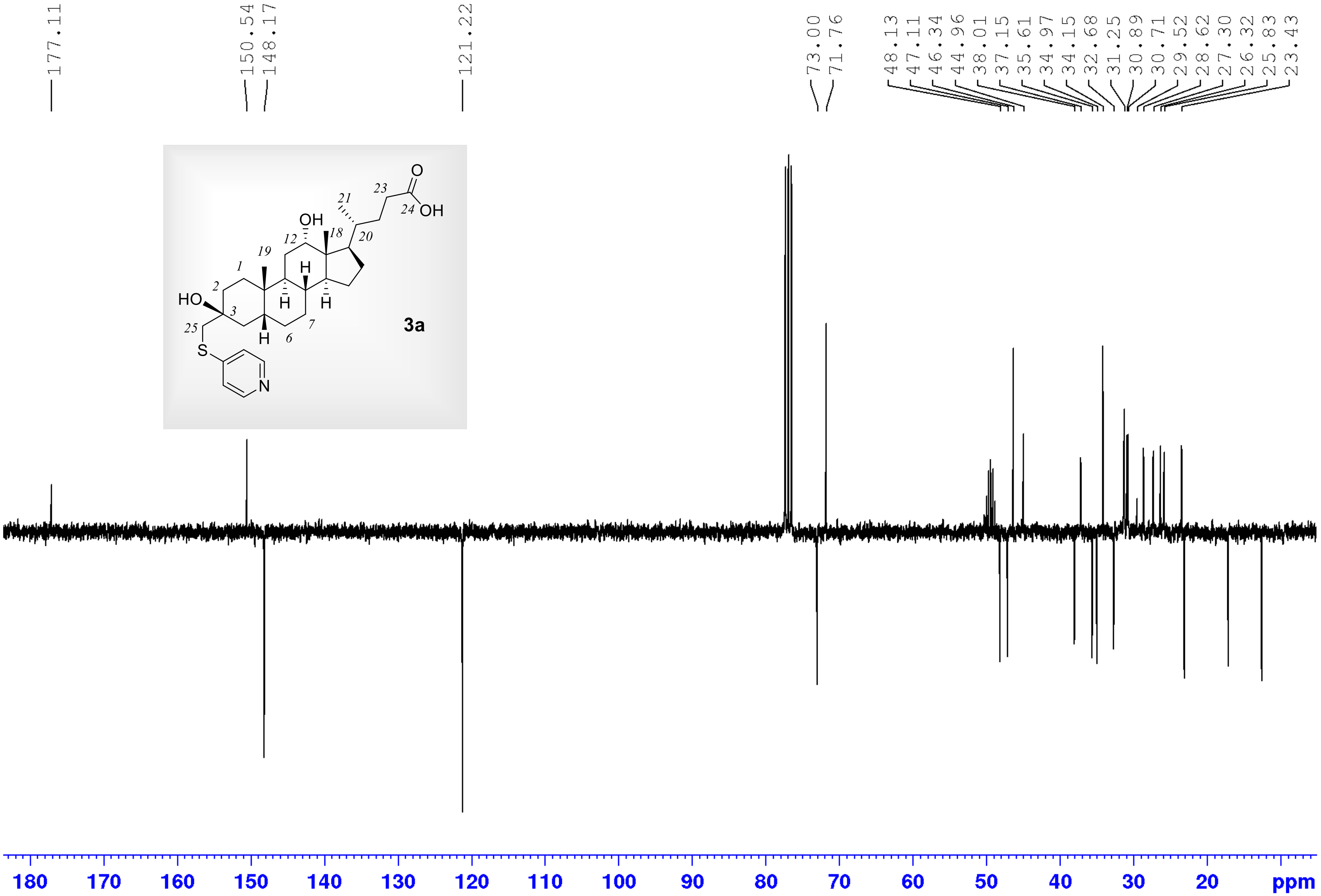

Spectrum of compound **3b**, ^{1}H NMR, 300MHz, $CDCl_3$

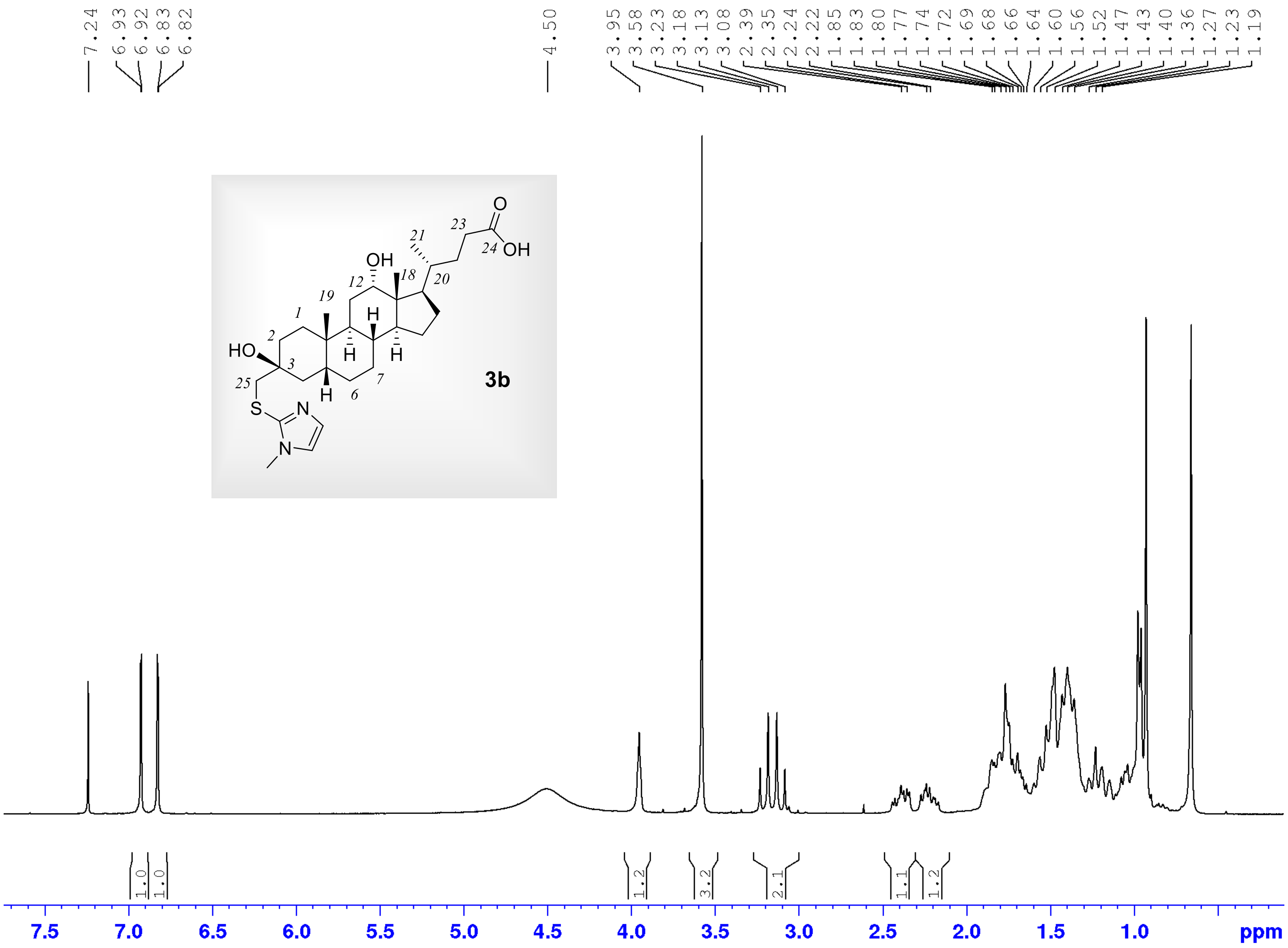

Spectrum of compound **3b**, ^{13}C NMR, 75MHz, $CDCl_3$+CD_3OD

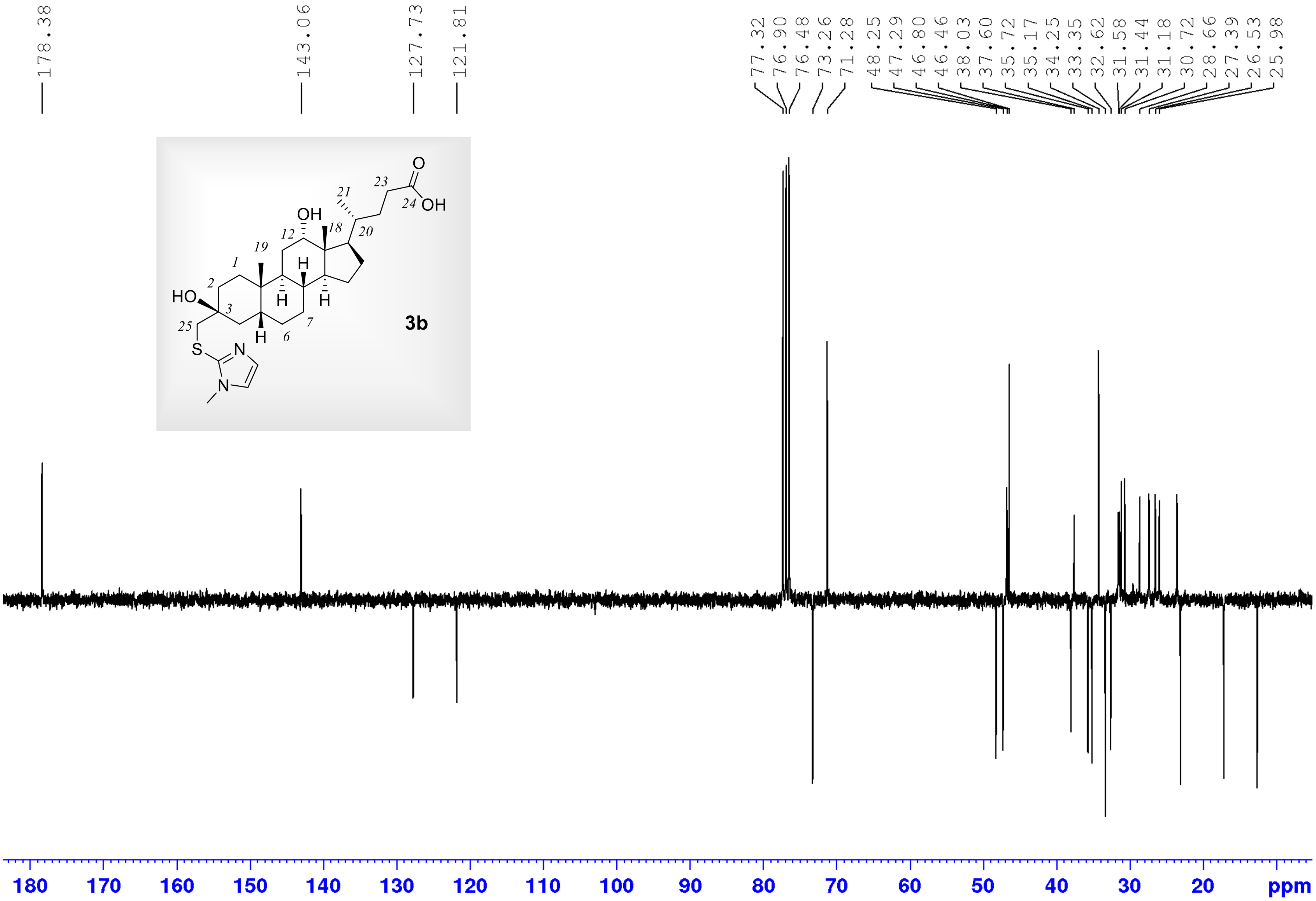

Spectrum of compound **3c,** ^{1}H NMR, 300 MHz, $CDCl_3$+CD_3OD

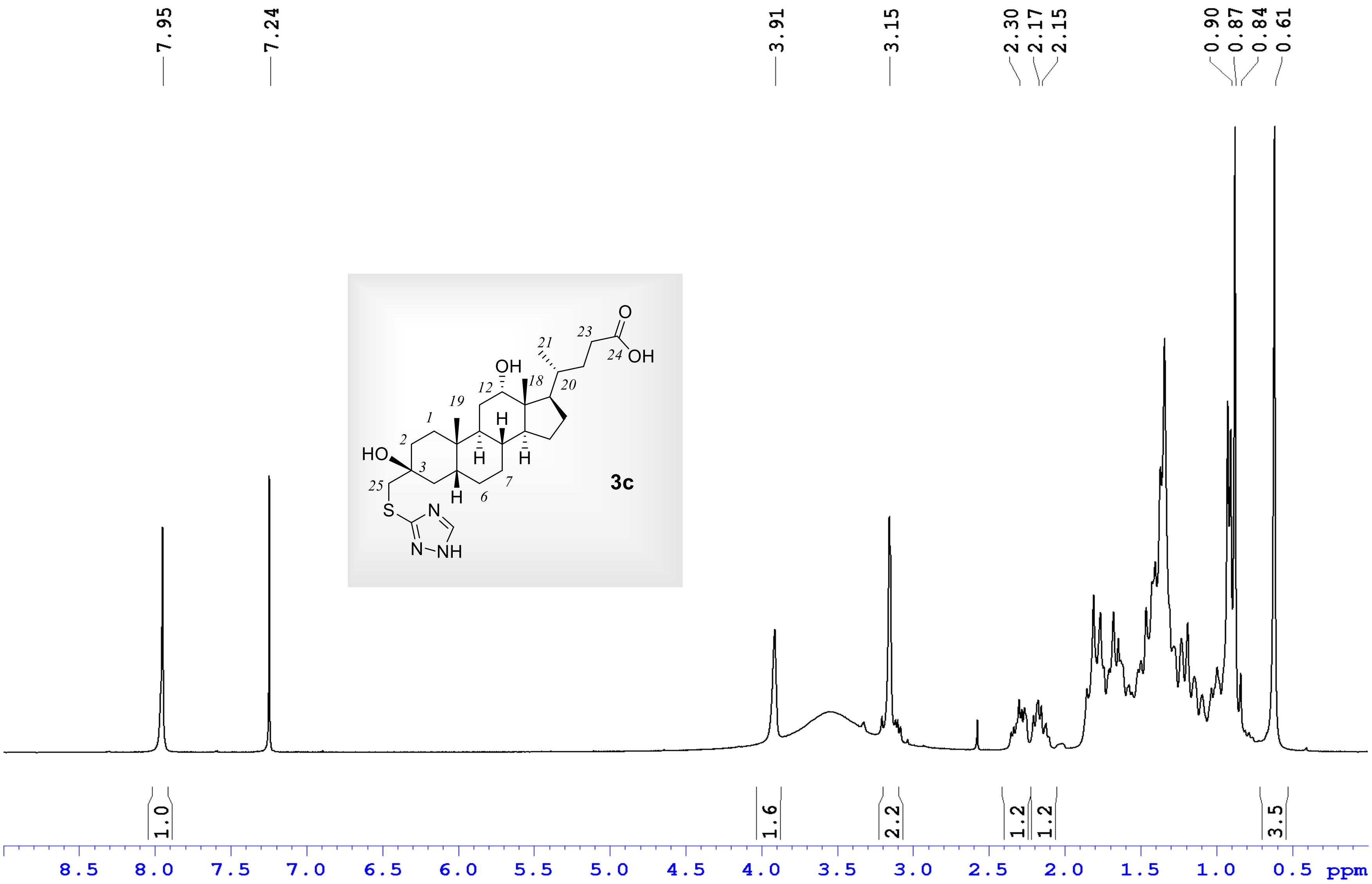

Spectrum of compound **3c,** ^{13}C NMR, 75 MHz, $CDCl_3$+CD_3OD

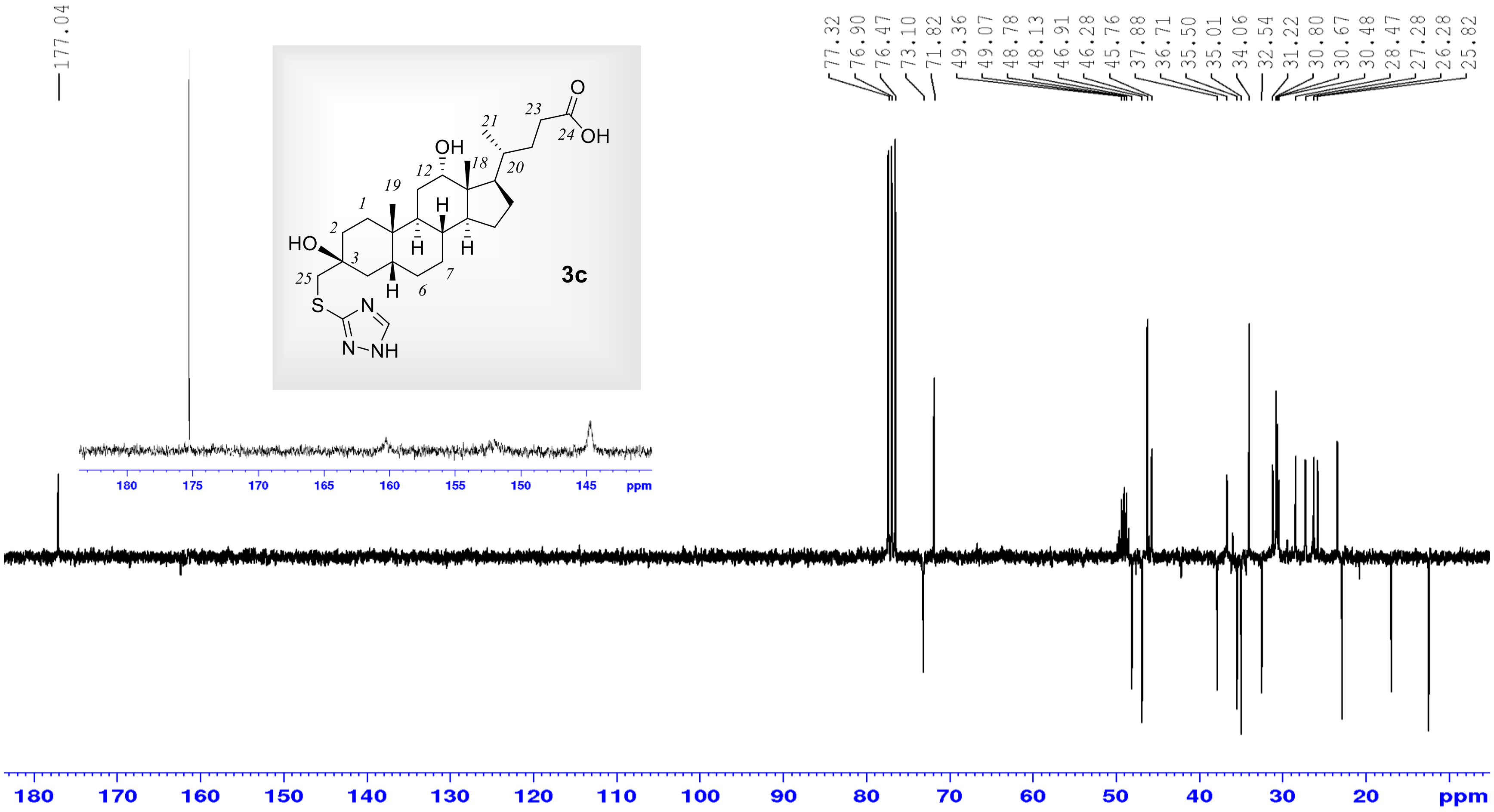

Spectrum of compound **3d,** ^{1}H NMR, 500 MHz, $CDCl_3$+CD_3OD

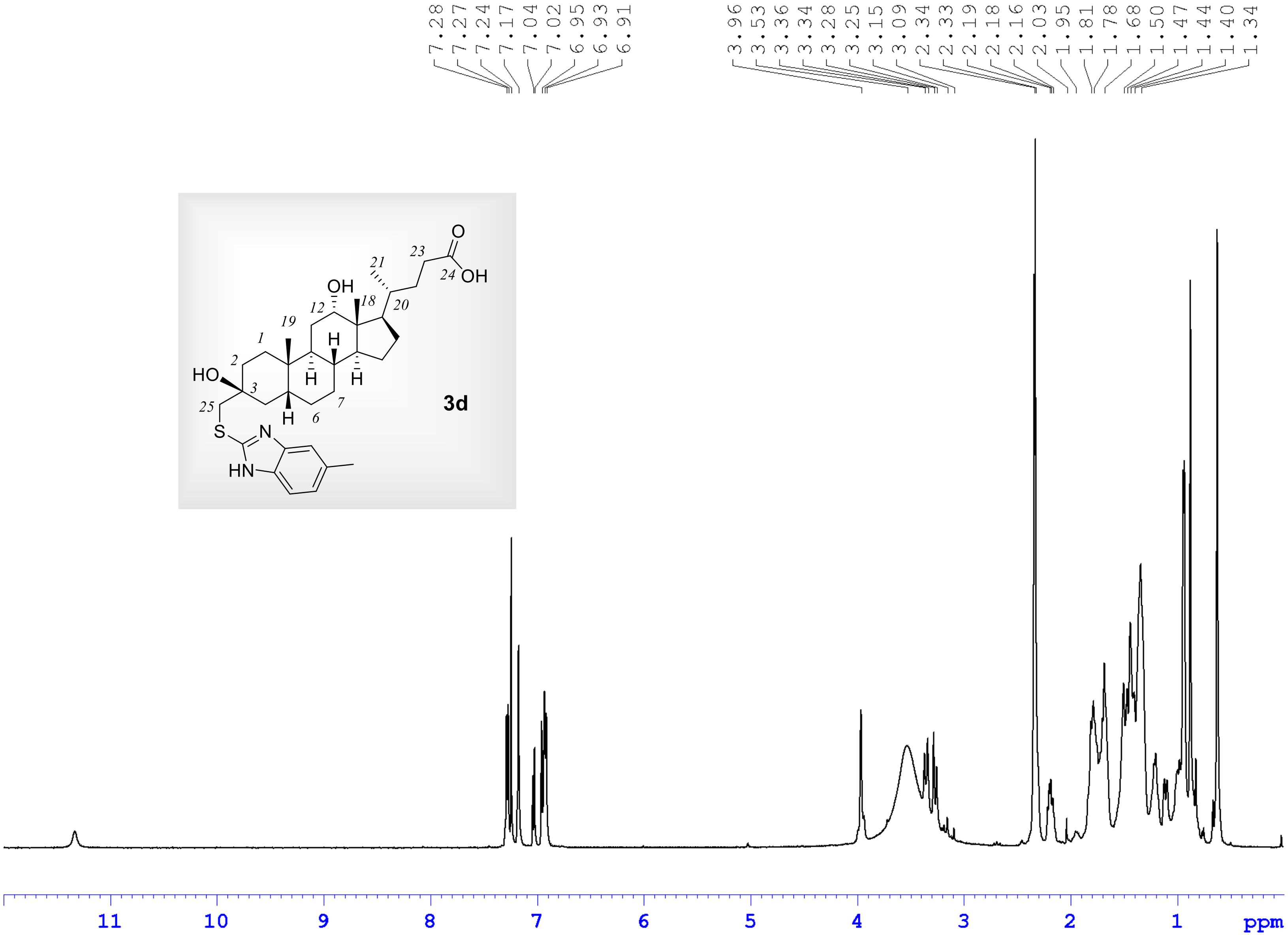

Spectrum of compound **3d,** ^{13}C NMR, 125 MHz, $CDCl_3$+CD_3OD

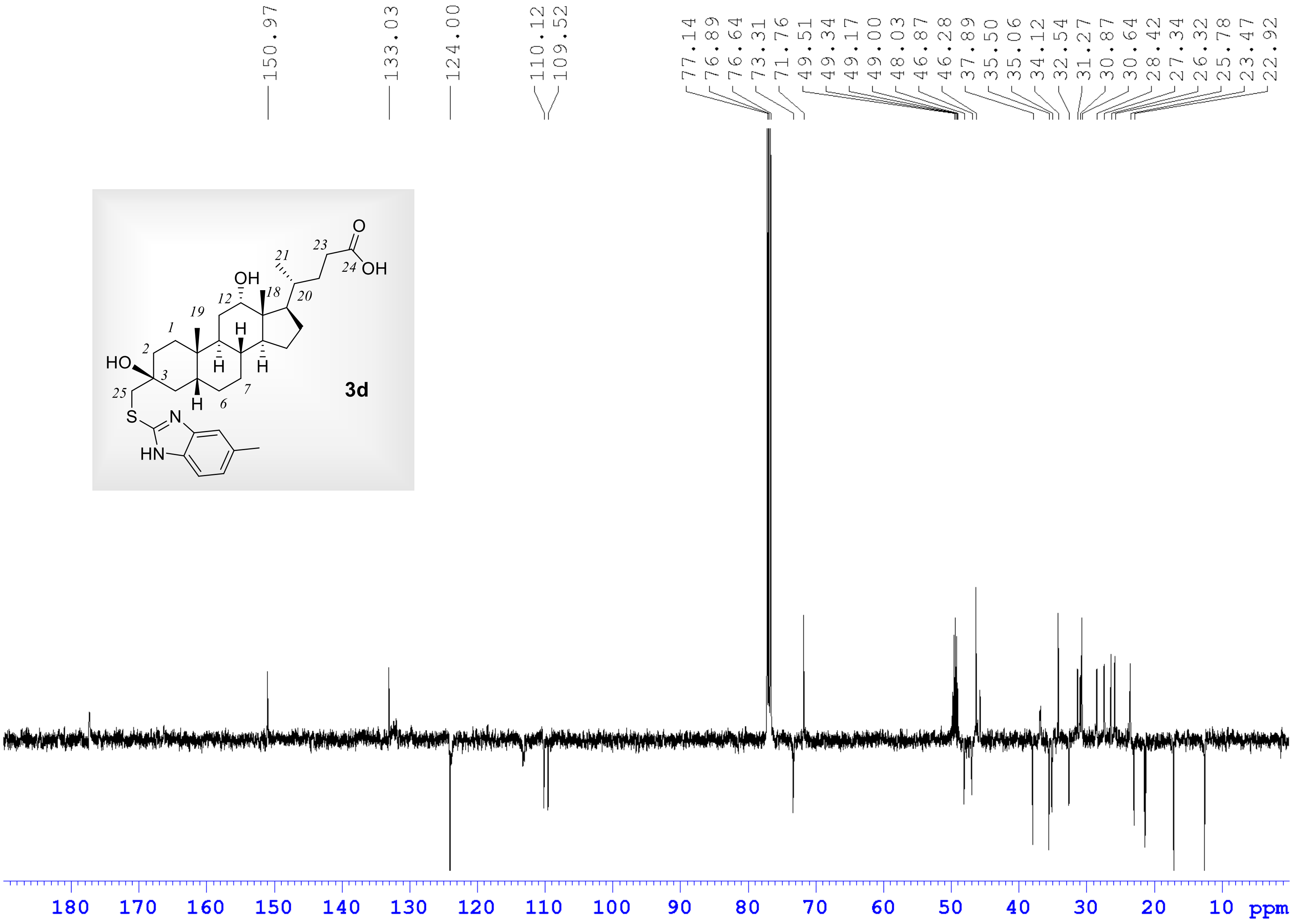

Spectrum of compound **3e,** ^{1}H NMR, 400 MHz, $CDCl_3$

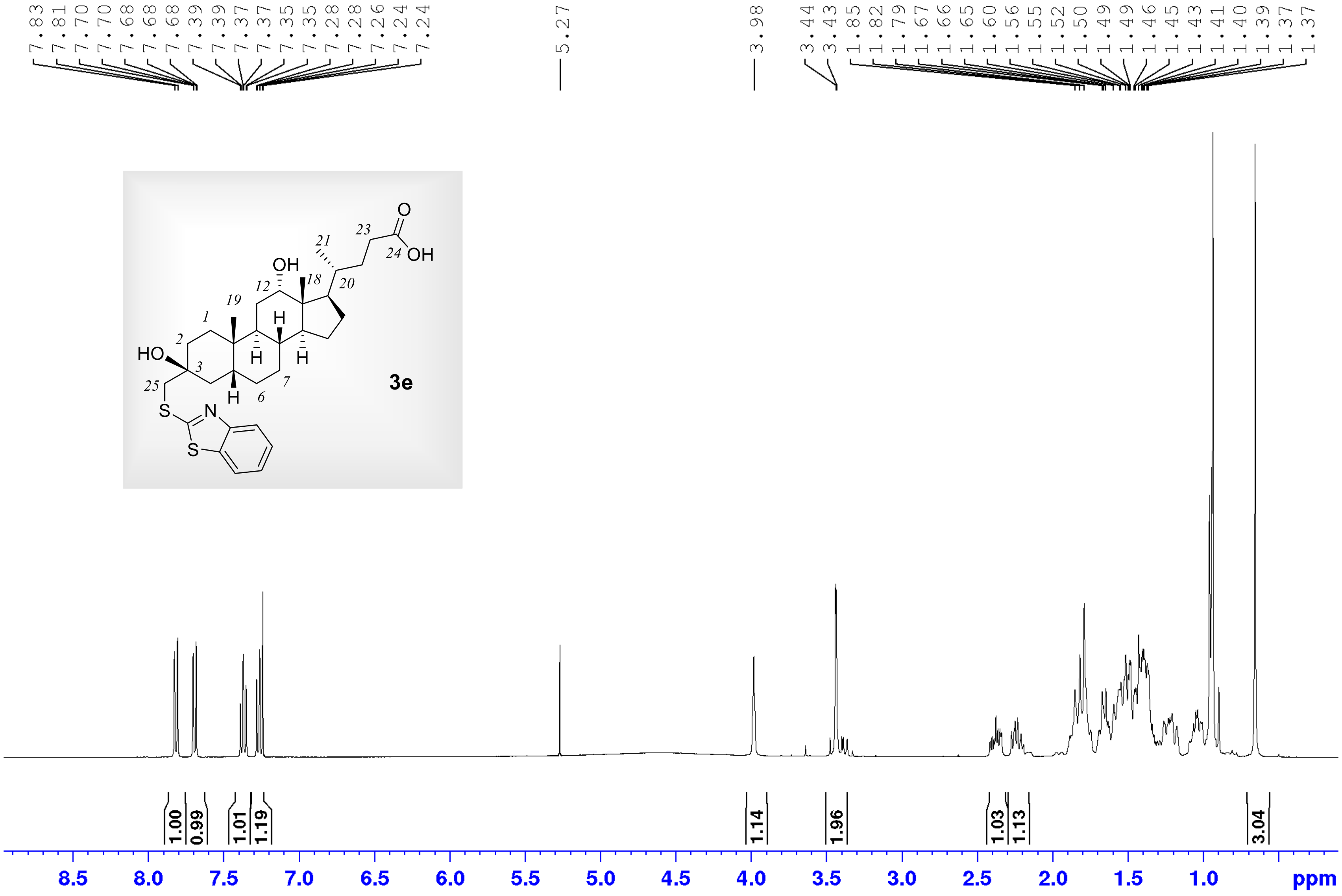

Spectrum of compound **3e,** ^{13}C NMR, 100 MHz, $CDCl_3$

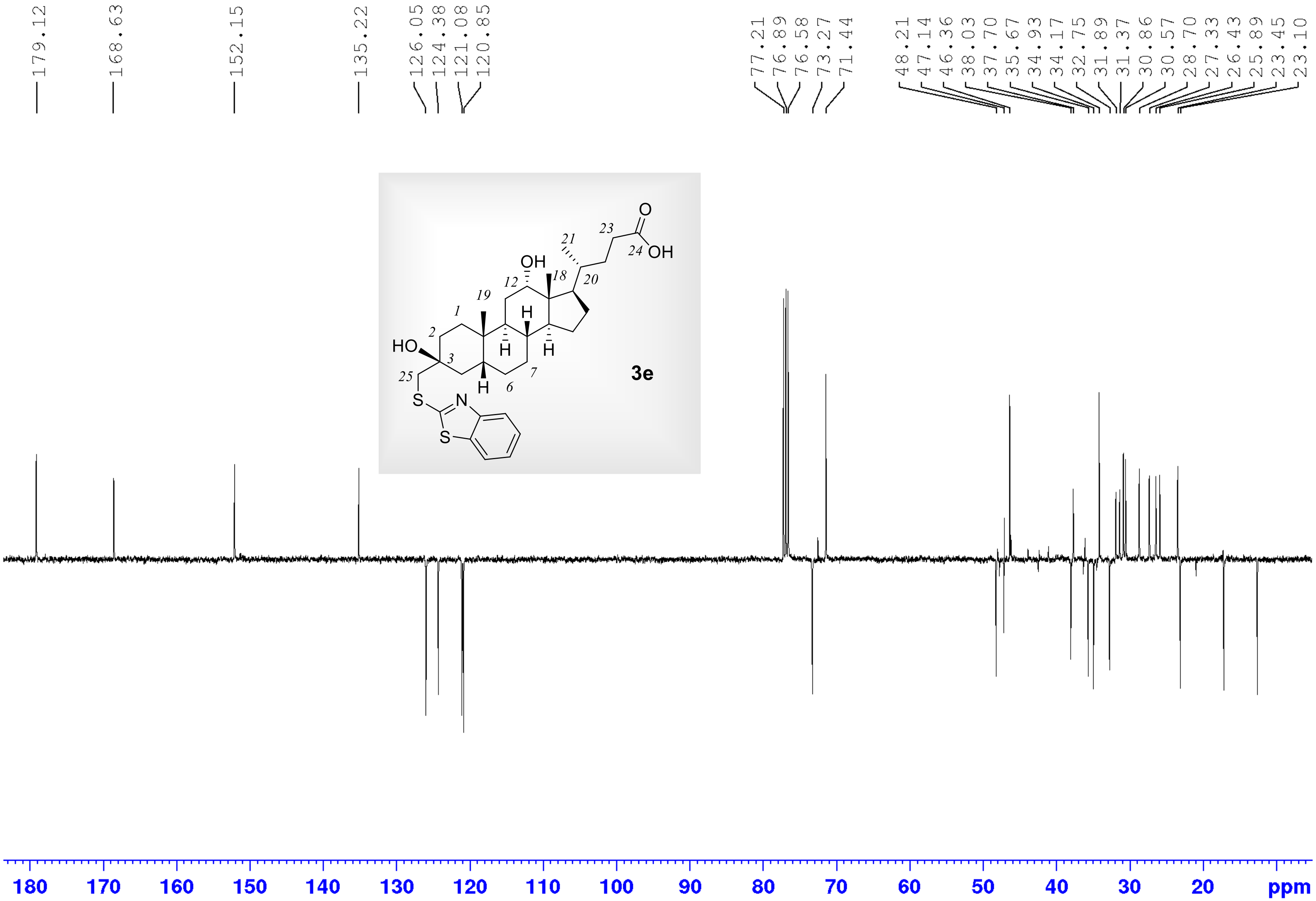

Spectrum of compound **4d,** ^{1}H NMR, 300 MHz, $CDCl_3$

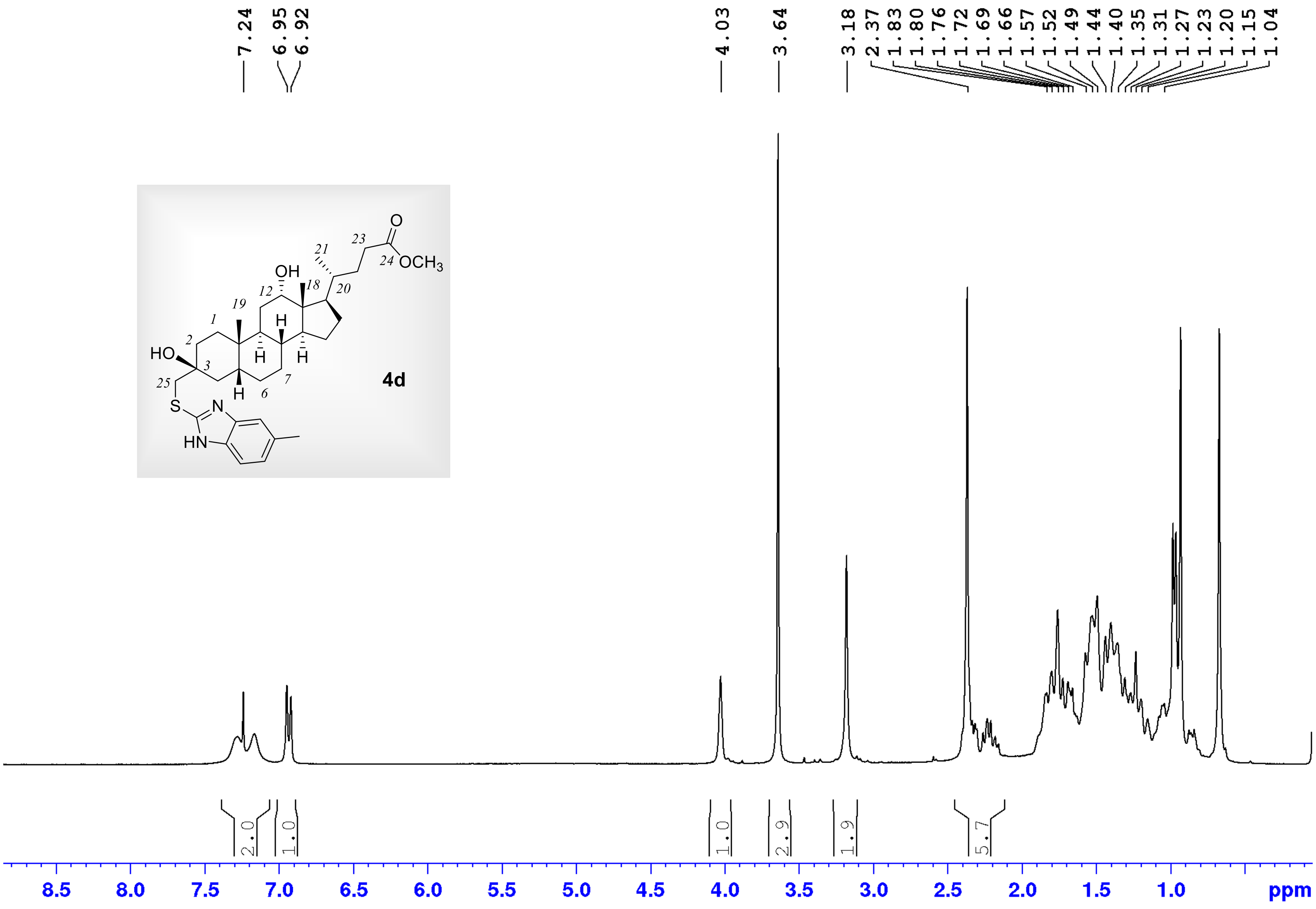

Spectrum of compound **4d,** ^{13}C NMR, 75 MHz, $CDCl_3$

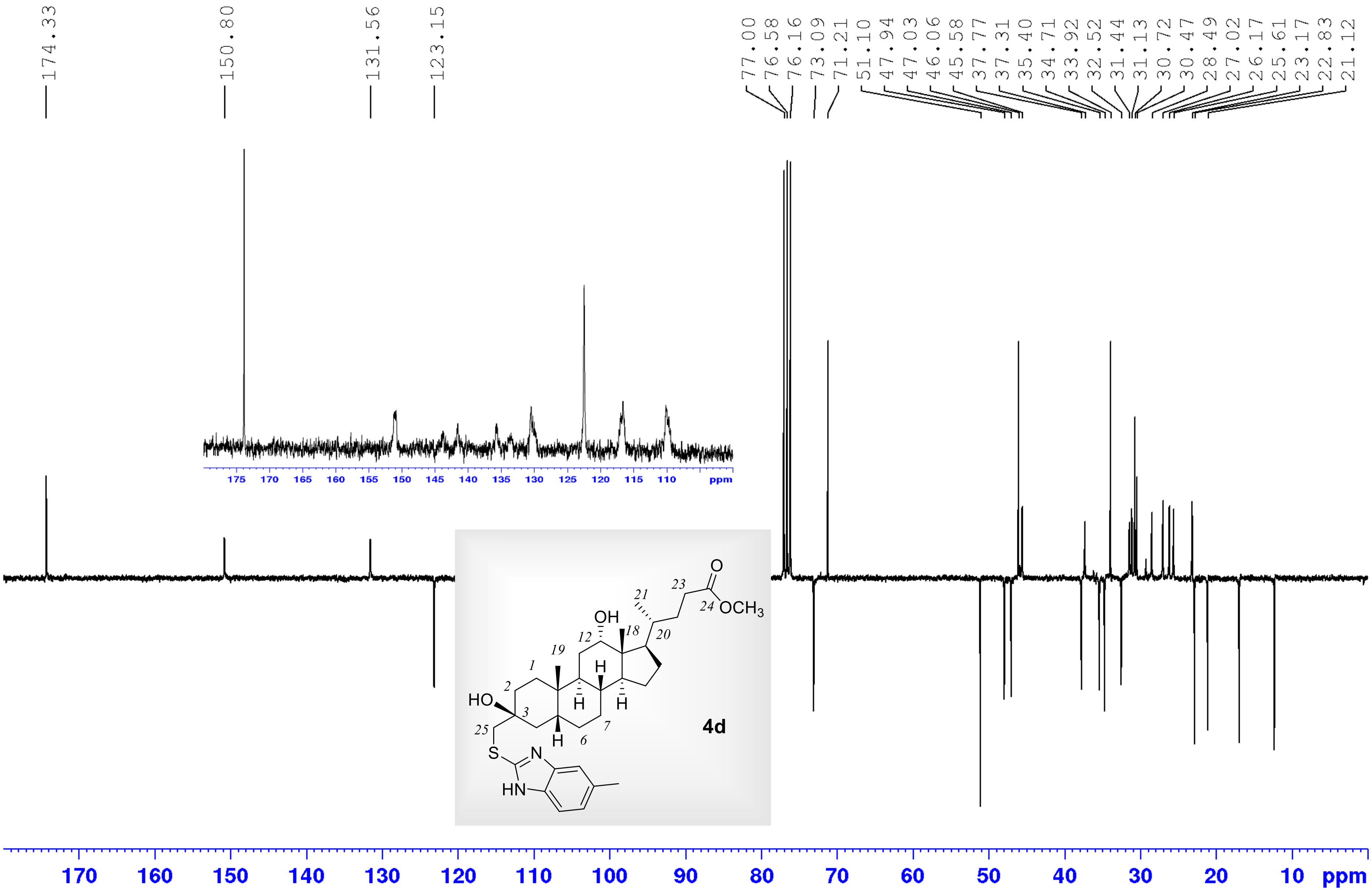

Spectrum of compound **4e,** ^{1}H NMR, 600 MHz, $CDCl_3$

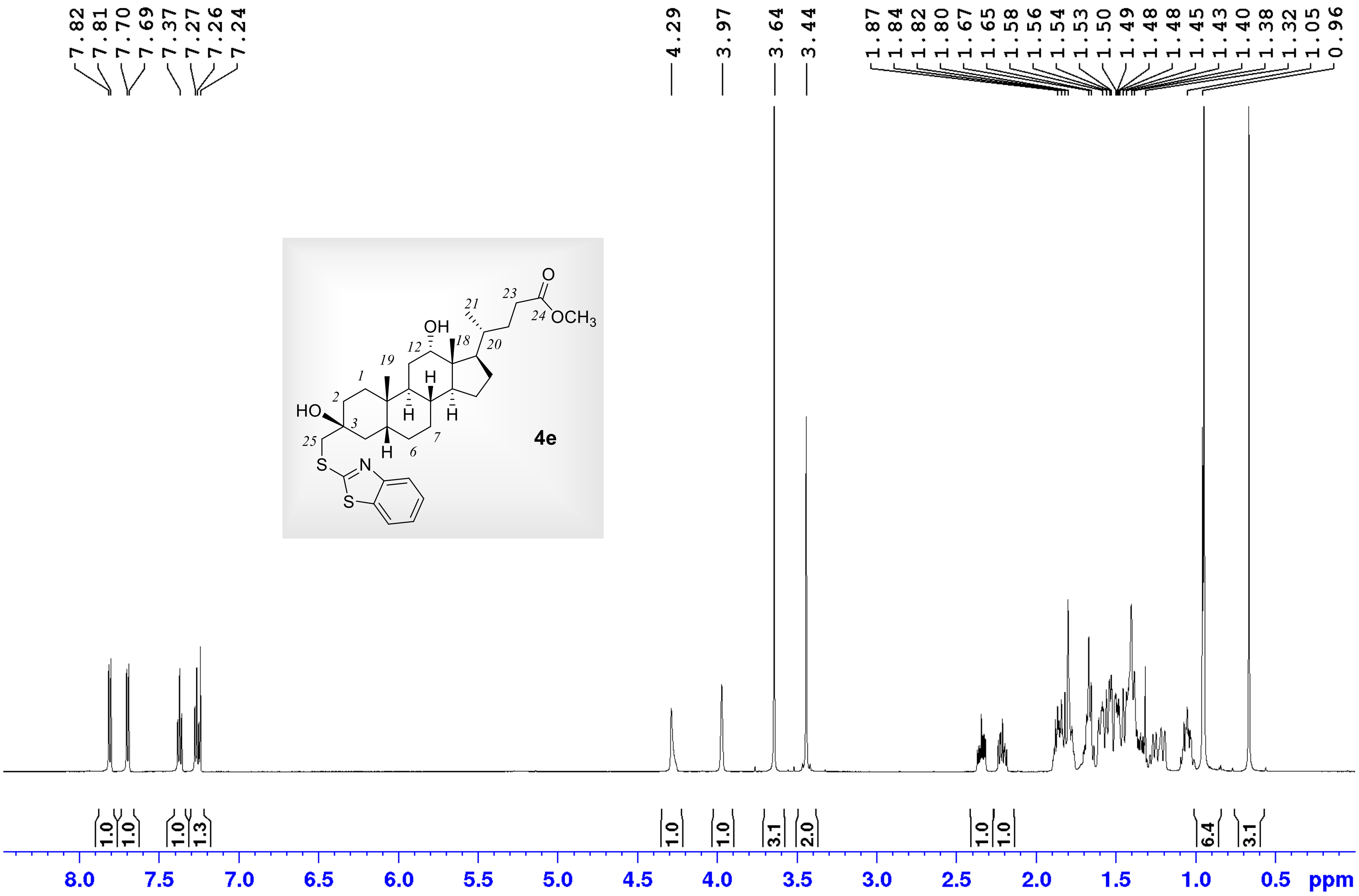

Spectrum of compound **4e,** ^{13}C NMR, 125 MHz, $CDCl_3$

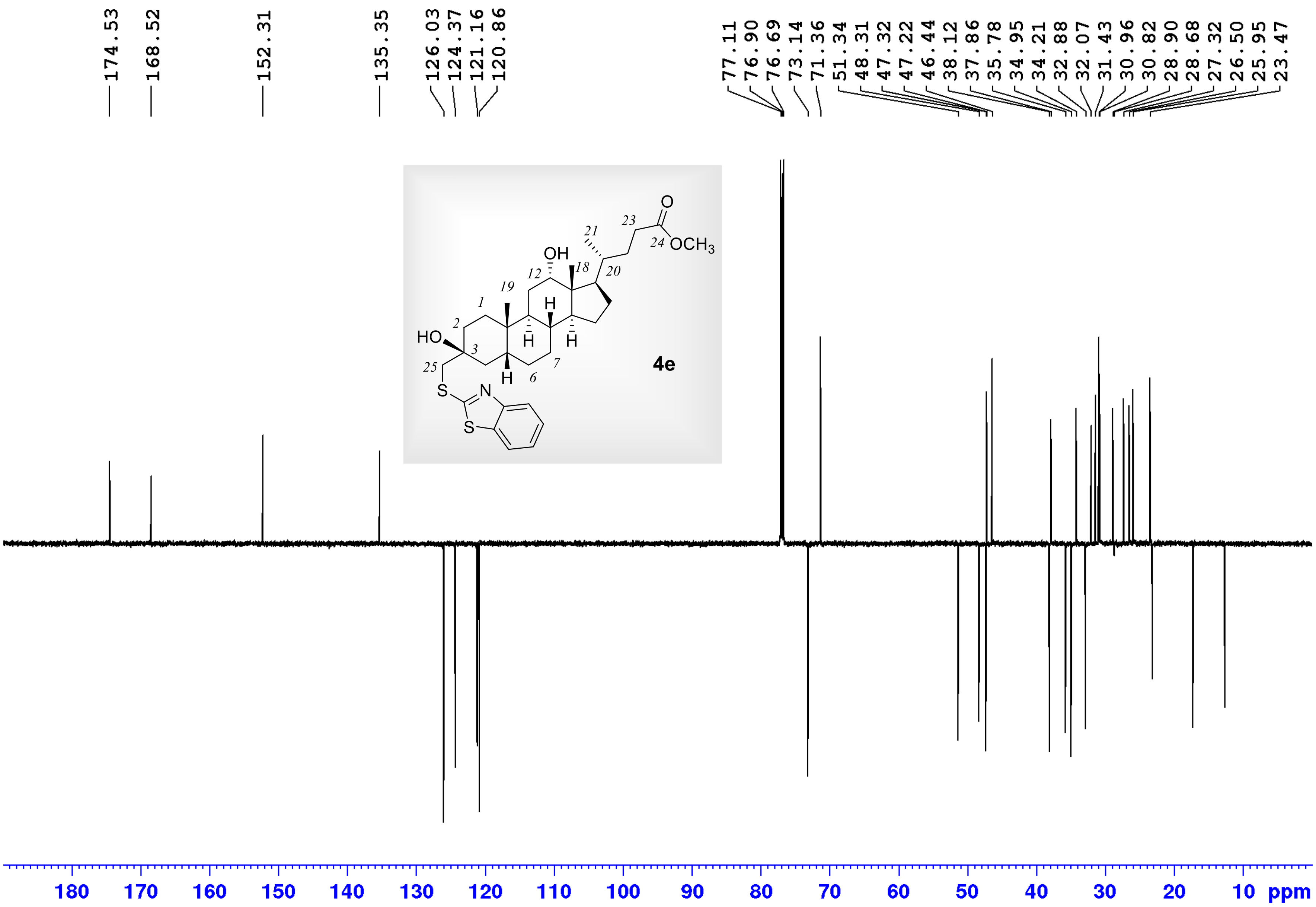

Spectrum of compound **4f,** ^{1}H NMR, 400 MHz, $CDCl_3$

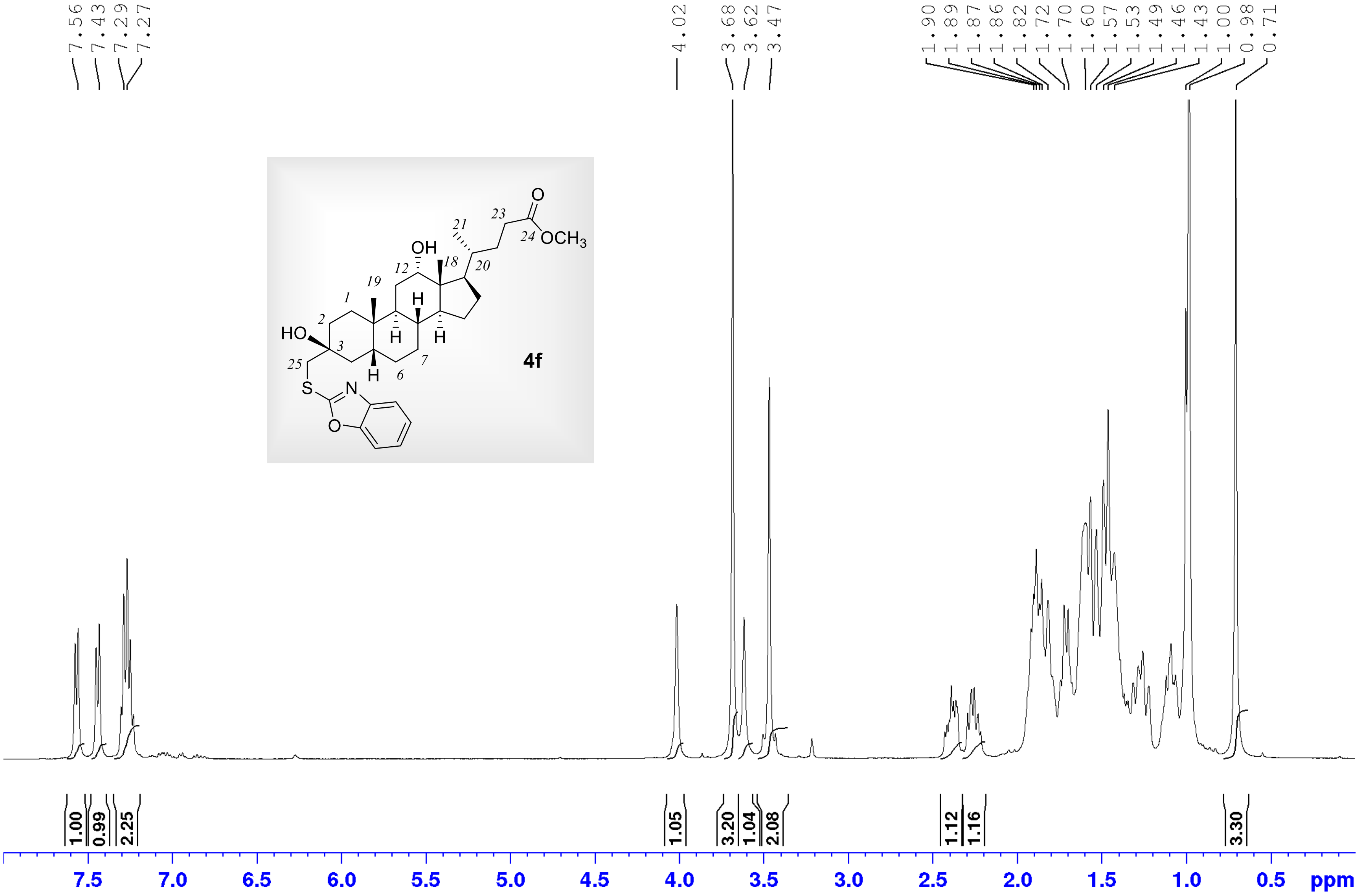

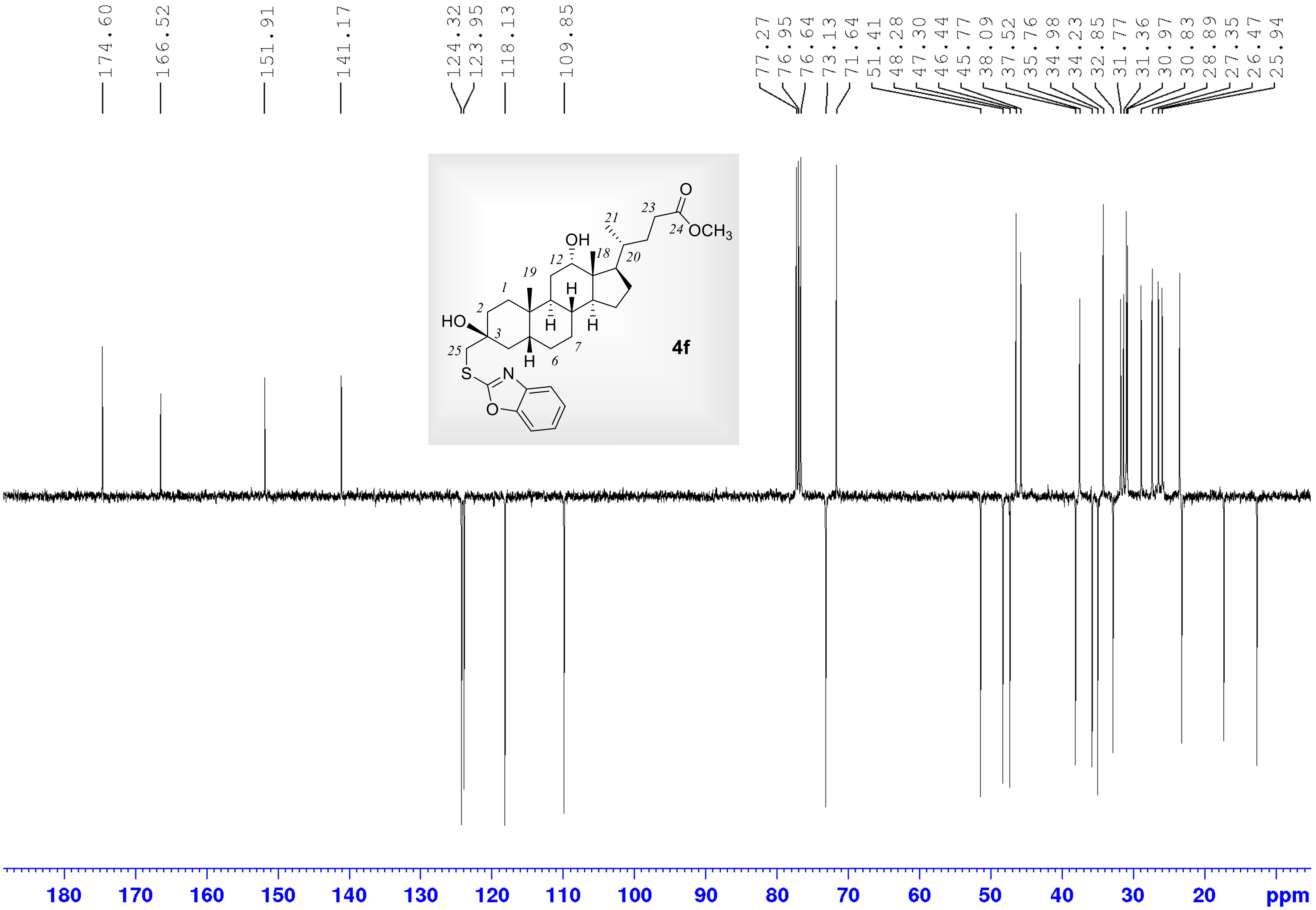
174.60
166.52
151.91
141.17
124.32
123.95
118.13
109.85
77.27
76.95
76.64
73.13
71.64
51.41
48.28
47.30
46.44
45.77
38.09
37.52
35.76
34.98
34.23
32.85
31.77
31.36
30.97
30.83
28.89
27.35
26.47
25.94
HO
OH
O
OCH3
S
N
O
H
4f
180
170
160
150
140
130
120
110
100
90
80
70
60
50
40
30
20
ppm

Table S1. The binding affinities as predicted by the scoring functions used to the catalytic TDP1 binding pocket and their measured IC_{50} values.

Ligand	ASP	ChemPLP	CS	GS	IC_{50}, μM
**3a**	45.0	55.8	22.9	48.5	22 ± 2
**3b**	45.7	60.4	20.3	48.8	30 ± 7
**3c**	47.6	50.5	16.2	54.1	32 ± 4
**3d**	43.3	63.5	24.4	58.1	25 ± 4
**3e**	47.2	62.4	22.2	52.3	14 ± 2
**4d**	44.0	63.8	27.6	57.3	0.94 ± 0.04
**4e**	45.1	60.5	24.4	52.5	0.63 ± 0.03
**4f**	43.6	59.4	25.1	53.3	1.8 ± 0.5

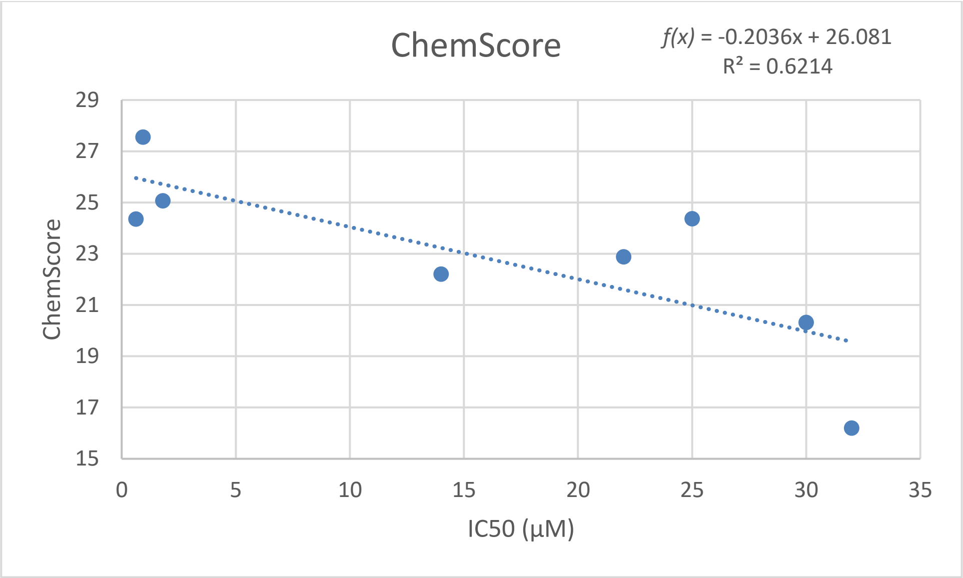

Figure S1. The correlation plot of measured IC_{50} values against their CS counterparts.

Table S2. The binding affinities as predicted by the scoring functions used to the catalytic TDP2 binding pocket and their measured IC_{50} values.

Ligand	**ASP**	**ChemPLP**	**CS**	**GS**	**IC_{50}, μM**
**3a**	36.0	54.7	21.7	50.1	N/A
**3b**	38.5	56.8	18.5	43.5	N/A
**3c**	33.8	52.6	14.4	49.3	N/A
**3d**	38.1	64.8	23.3	56.2	250
**3e**	39.0	62.4	21.3	53.9	76
**4d**	38.9	65.1	26.0	57.1	218
**4e**	39.6	62.6	24.0	55.1	N/A
**4f**	39.9	61.3	23.8	55.0	N/A

Table S3. The molecular descriptors and their corresponding Known Drug Indexes 2a and 2b ($KDI_{2a/2b}$).

	RB	MW	HD	HA	Log P	PSA	KDI_{2A}	KDI_{2B}
**3a**	9	515.8	3	6.45	5.4	92.6	4.12	0.07
**3b**	9	518.8	3	5.95	5.6	96.4	4.07	0.06
**3c**	9	505.7	4	7.45	4.2	125.6	3.74	0.05
**3d**	9	568.8	4	5.95	6.5	108.1	3.24	0.01
**3e**	9	571.8	3	5.95	6.6	93.4	3.65	0.02
**4d**	9	582.8	3	5.95	7.0	94.1	3.55	0.01
**4e**	9	585.9	2	5.95	7.1	79.4	3.75	0.01
**4f**	9	569.8	2	6.45	6.7	88.7	3.82	0.02
**TDP1 - R^2**	X	0.686	0.602	0.171	0.685	0.550	0.025	0.435
**TDP2 - R^2**	X	0.021	0.412	X	0.036	0.452	0.658	0.980

Table S4. Definition of lead-like, drug-like and Known Drug Space (KDS) in terms of molecular descriptors. The values given are the maxima for each descriptor for the volumes of chemical space used.

	**Lead-like Space**	**Drug-like Space**	**Known Drug Space**
Molecular weight (g mol^{-1})	300	500	800
Lipophilicity (Log P)	3	5	6.5
Hydrogen bond donors (HD)	3	5	7
Hydrogen bond acceptors (HA)	3	10	15
Polar surface area ($Å^2$) (PSA)	60	140	180
Rotatable bonds (RB)	3	10	17

HPLC analyses for compound **3a-e, 4d-f** and 3α,12α-*bis*-methoxy deoxycholic *para*-bromoanilide (compound **A**) and 3α-benzyloxy deoxycholic tryptamide (compound **B**)
HPLC analyses were carried out on a MilichromA-02, using a ProntoSIL 120-5-C18 AQ column (BISCHOFF, 2.0 × 75 mm column, grain size 5.0 μm). The mobile phase was Millipore purified water with 0.1% trifluoroacetic acid (eluent **A**) at a flow rate of 150 μL/min at 35°C with UV detection at 210, 220, 240, 260 and 280 nm. A typical run time was 25 min with a gradient of 30–100% acetonitrile (eluent **B**)

Eluent A+B**,** flow 500 μL/min								
Volume , μL	500	500	1000	1500	2000	2500	3000	3500
eluent A, %	100	70	50	30	10	10	0	0
eluent B, %	0	30	50	70	90	90	100	100

Compound	Log P (*in silico*)	HLPC, *time*
**3a**	5.4	9 min 53 s (1483 μL)
**3b**	5.6	9 min 47 s (1469 μL)
**3d**	6.5	10 min 46 s (1614 μL)
**4d**	7.0	12 min 20 s (1849 μL)
**3c**	4.2	12 min 20 s (1950 μL)
**3e**	6.6	14 min 45 s (2212 μL)
**4f**	6.7	16 min 28 s (2471 μL)
**4e**	7.1	17 min 08 s (2569 μL)
3α-benzyloxy deoxycholic tryptamide (compound **B**)	8.0	17 min 40 s (2649 μL)
3α,12α-*bis*-methoxy deoxycholic *para*-bromoanilide (compound **A**)	7.4	20 min 41 s (3102 μL)

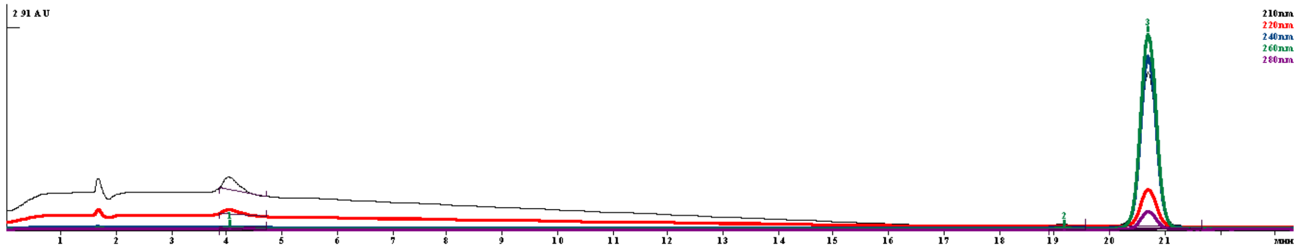

compound **A**

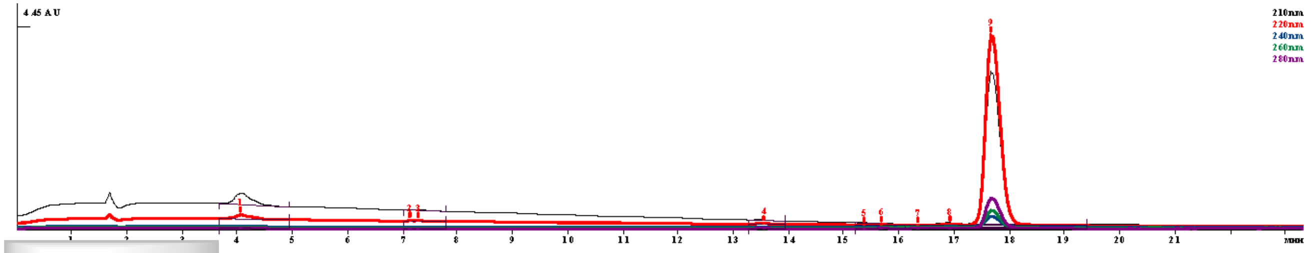

compound **B**

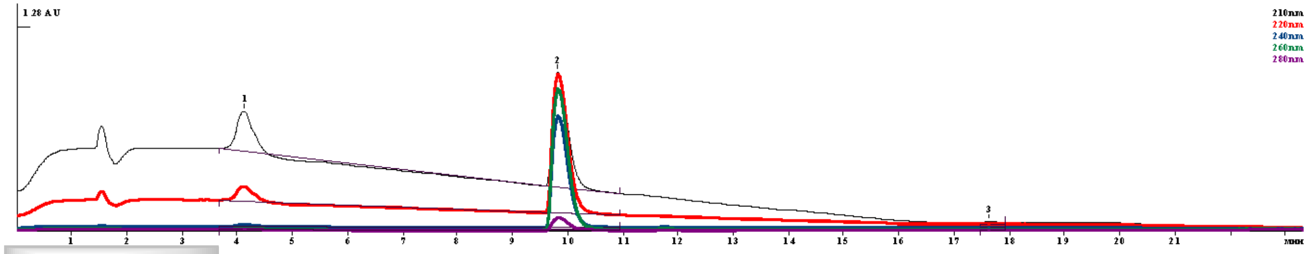

compound **3b**

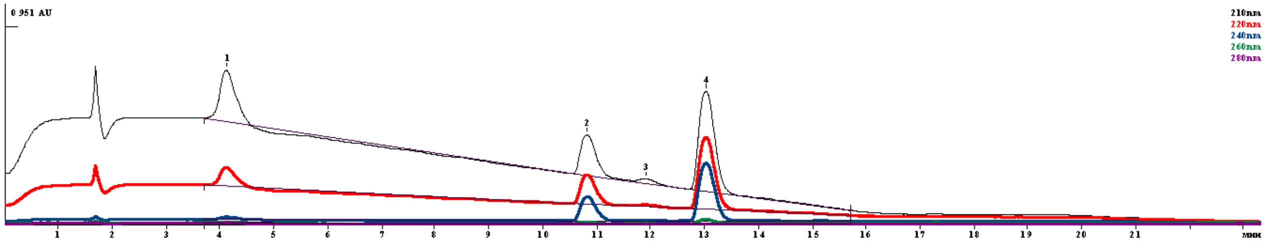

compound **3c**

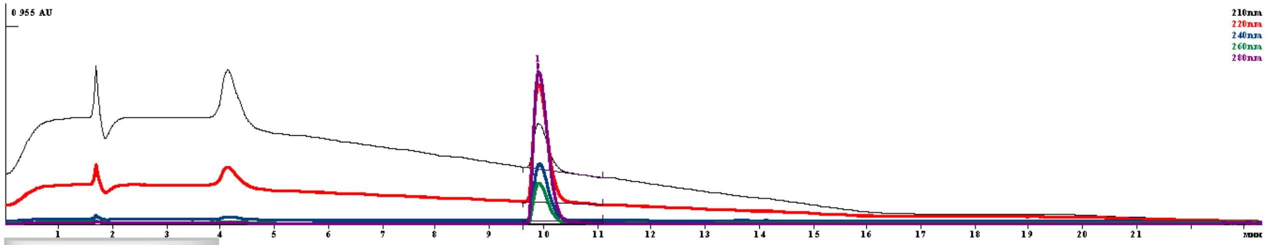

compound **3a**

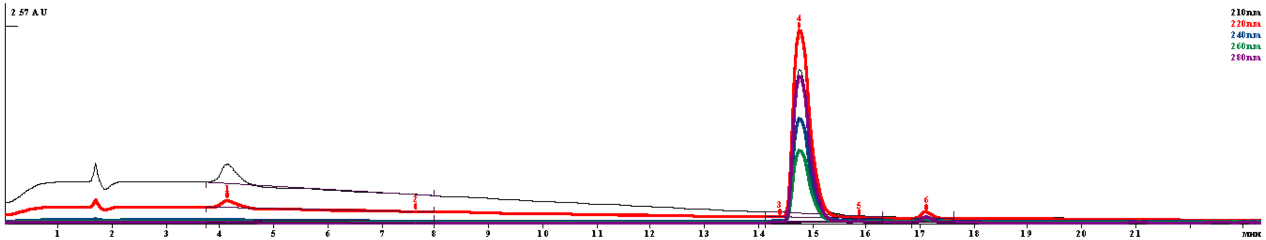

compound **3e**

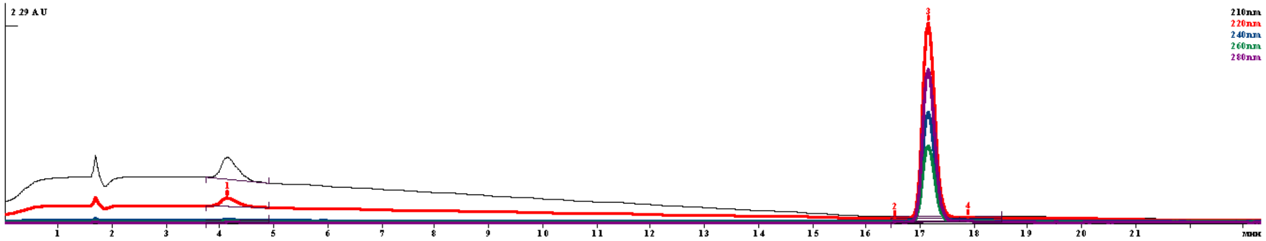

compound **4e**

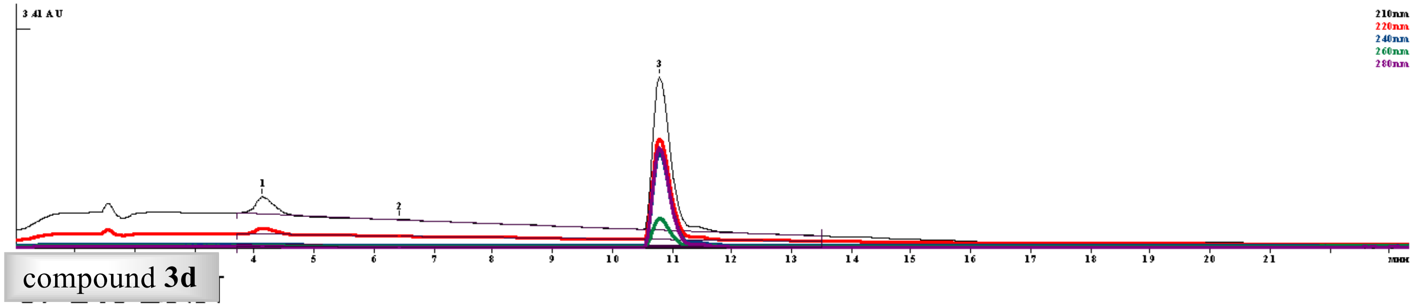

compound **3d**

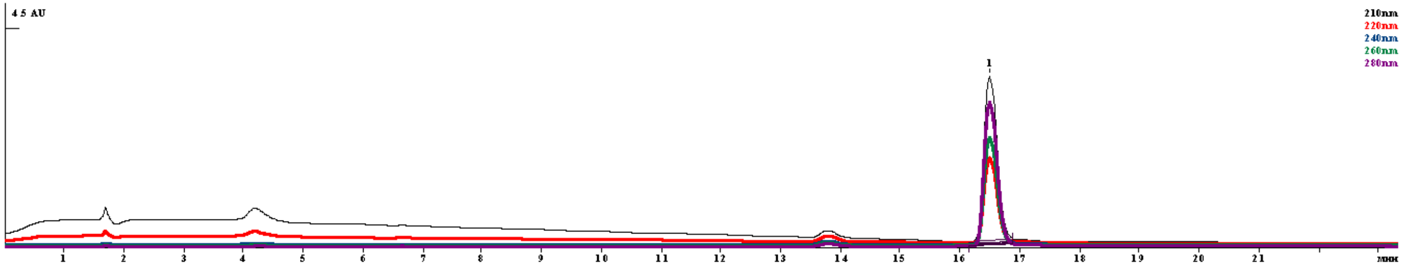

compound **4f**

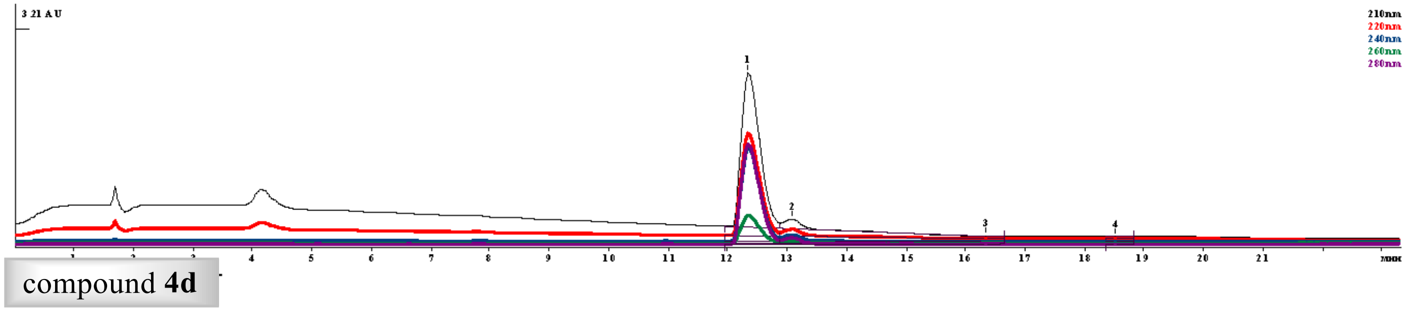

compound **4d**
